# Molecular Bases and Genetic Design of Rice Disease Resistance for Optimized Yield and Sustainable Agriculture

**DOI:** 10.1002/advs.77719

**Published:** 2026-09-16

**Authors:** Xinyue Hou, Yuanjiang Cui, Chaoqing Ding, Qian Qian, Deyong Ren

**Affiliations:** ^1^ State Key Laboratory of Rice Biology and Breeding China National Rice Research Institute Hangzhou China

**Keywords:** breeding strategy, disease resistance, genetic improvement, high yield, immune mechanism, rice

## Abstract

Rice diseases continue to undermine yield stability and threaten the sustainability of rice production. The central challenge is therefore not simply to maximize immune activation, but to identify genetic interventions that remain effective across diverse pathogen races and environmental conditions without imposing excessive penalties on growth or yield. Here, we synthesize the molecular basis of rice immunity from a design‐oriented perspective. We first examine cell‐surface pattern‐recognition receptors and intracellular nucleotide‐binding leucine‐rich repeat receptors, and then assess the shared signaling hubs and defence outputs that connect pathogen perception to antimicrobial responses. Rather than treating these components as equivalent breeding targets, we compare their translational potential according to resistance spectrum, anticipated durability, tunability, pleiotropic risk, and the strength of field evidence. We further discuss breeding strategies based on receptor engineering, editing of susceptibility genes and cis‐regulatory elements, post‐translational motif engineering, pathogen‐inducible and upstream open reading frame‐mediated regulation, resistance‐gene stacking and artificial intelligence‐assisted prediction. We argue that rational resistance design in rice should move beyond constitutive immune activation toward allele‐specific, quantitative, spatially restricted and infection‐responsive regulation. Integrating mechanistic insights with precision genome editing, accelerated breeding and responsible deployment offers a practical route to durable, yield‐compatible disease resistance while reducing dependence on chemical control.

## Graphical abstract

This study integrates rice immune perception, signal transduction, and resistance output into a multi‐omics framework. By combining high‐throughput sequencing, phenomics, and AI‐assisted design with marker‐assisted selection, gene editing, and rapid domestication, we aim to accelerate the precise breeding of disease‐resistant rice varieties by systematically identifying and deploying key resistance‐associated genes.

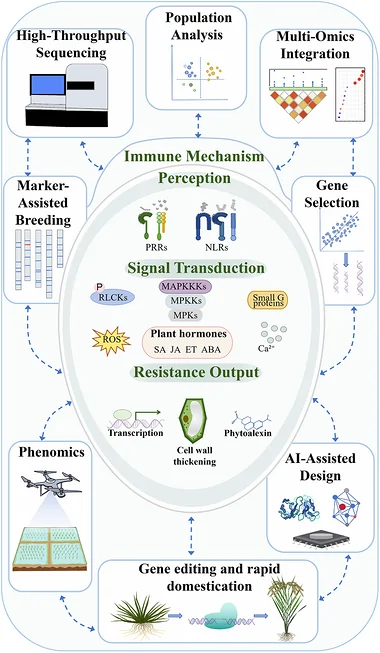

## 1 Introduction

Rice diseases, including bacterial blight, rice blast, viral diseases, and emerging pathogen threats, remain major constraints to yield stability and food security [1, 2, 3]. Their impacts are exacerbated by pathogen evolution, transboundary dispersal, and climate‐driven changes in cropping environments. Chemical control and conventional resistance breeding provide only partial solutions: pesticide use imposes economic and environmental costs, single resistance genes can be rapidly overcome, and constitutive immune activation often incurs growth‐defence trade‐offs. Disease‐resistance breeding must therefore deliver broad, durable, and environmentally stable protection without compromising yield.

Advances in rice genomics and immunity now enable a transition from empirical resistance‐gene deployment to rational immune design [4, 5]. Key components‐including pattern‐recognition receptors, NLRs, shared signaling modules, hormonal and reactive oxygen species networks, defensive metabolites, and host targets of pathogen virulence factors‐can increasingly be manipulated through pangenomics, natural‐variation mining, genome editing, multiplex engineering, and high‐throughput phenotyping. The central challenge is to identify targets that combine mechanistic relevance with tunability, durability, and agronomic safety [6, 7].

In this Review, we organize recent advances in rice immunity around the major challenges that constrain the durability and agronomic value of disease resistance. We examine how pathogen recognition, immune signaling, defence execution, and pathogen counter‐defence collectively shape resistance spectrum, durability, environmental robustness, and growth‐defence trade‐offs. On this basis, we assess the translational potential of different genetic strategies according to their capacity to overcome these constraints, as well as their technical feasibility, pleiotropic effects, risk of pathogen adaptation, and degree of field validation (Figure 1 and Table S1). We focus on the editing of susceptibility genes and cis‐regulatory elements, quantitative or inducible tuning of immune responses, resistance‐gene stacking, the exploitation of microbiome‐associated resistance, and the validation of performance across environments. This framework aims to guide the development of durable disease resistance while safeguarding yield and promoting agricultural sustainability.

**FIGURE 1:**
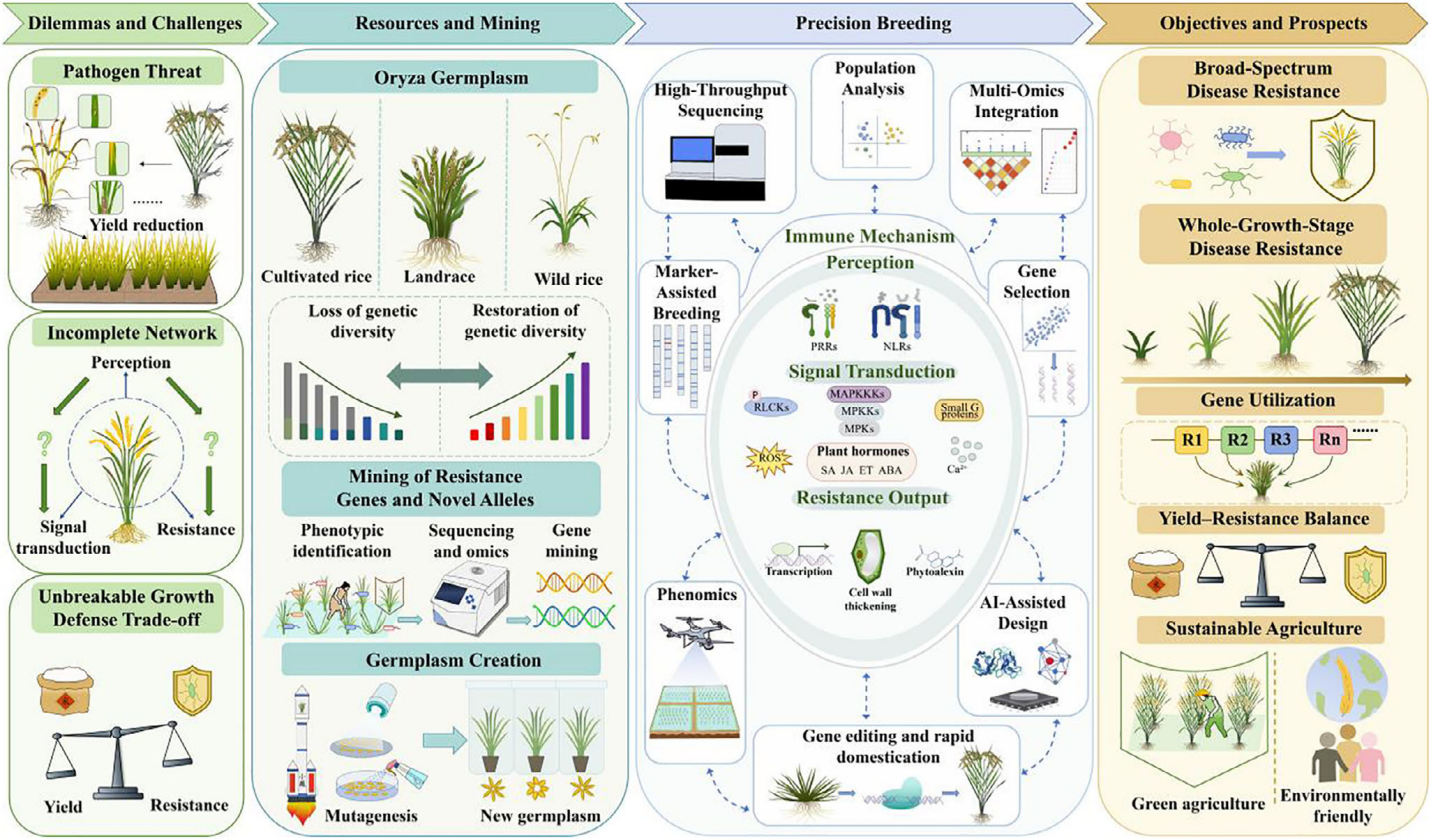
Optimizing disease resistance and yield via integrating genetic resource utilization with molecular design for sustainable agriculture.

Rice faces multiple challenges in achieving both disease resistance and stable yield in natural environments, including pathogen pressure, an incomplete understanding of the molecular mechanisms underlying rice immunity, and the difficulty of overcoming the trade‐off between yield and disease resistance (green section). Germplasm resources are the “chips” of agriculture, and their extensive collection provides the foundation for disease‐resistance breeding. Current rice germplasm resources mainly include cultivated rice, landraces and wild rice. However, during the gradual domestication of wild rice, genetic diversity has been progressively lost. Disease‐resistance genes and novel allelic variants can be identified through approaches such as phenotypic evaluation and gene sequencing, whereas new germplasm can be created through mutagenesis (cyan section). The integration of artificial intelligence‐assisted prediction and high‐throughput phenomics can accelerate the dissection of rice immune mechanisms. Meanwhile, the application of modern biotechnologies to exploit diverse germplasm resources can help overcome the subjectivity and efficiency bottlenecks of conventional breeding. Together, these strategies will accelerate the development of rice varieties that combine full‐growth‐stage disease resistance, broad‐spectrum resistance and stable yield, thereby helping to balance yield and disease resistance and promote sustainable agriculture (blue and orange sections).

## 2 The Rice Immune System: An Invisible Battlefield

In response to pathogen invasion, rice has evolved a multilayered immune system. The zig‐zag model provides a useful framework for understanding this process. Pattern recognition receptor (PRR) first recognize pathogen‐associated molecular patterns (PAMPs) and host‐derived danger signals, thereby activating pattern‐triggered immunity (PTI). To overcome PTI, pathogens deliver effectors into host cells, causing effector‐triggered susceptibility (ETS). In turn, intracellular resistance proteins recognize these effectors, or the host perturbations they induce, and activate effector‐triggered immunity (ETI), which is frequently accompanied by the hypersensitive response (HR). Pathogens may escape ETI through effector diversification, thereby driving continuous host‐pathogen coevolution [8, 9]. This model depicts plant immunity as a dynamic continuum shaped by an evolutionary arms race between plants and pathogens. Importantly, PTI and ETI should not be viewed as discrete, independent defence layers; rather, they share extensive downstream signaling components and often function in a coordinated and mutually reinforcing manner [10, 11]. As this section focuses on the rice immune system, the following section specifically discusses PTI and ETI pathways in this crop.

### 2.1 The Sentinels of Immunity: PRR Receptor Proteins

#### 2.1.1 Overview of PRR Proteins

As the first layer of plant immune surveillance, PRRs function as cell‐surface sentinels that perceive invading pathogens and activate downstream defence signaling. Plant PRRs are broadly classified into receptor‐like kinases (RLKs) and receptor‐like proteins (RLPs). RLKs generally contain an extracellular ligand‐binding domain, a transmembrane region and an intracellular kinase domain, whereas RLPs lack cytoplasmic kinase activity and therefore depend on co‐receptors for signal transduction. Through diverse extracellular domains, including leucine‐rich repeat, lectin and lysin‐motif domains, PRRs recognize a wide range of extracellular immunogenic signals [12]. Efficient PRR‐mediated perception is especially critical during the early stages of pathogen attack, when different invaders adopt distinct entry strategies. *Xanthomonas oryzae* pv*. Oryzae (Xoo)* enters rice tissues through stomata or wounds and spreads intercellularly [13], whereas *Magnaporthe oryzae* forms appressoria and penetration pegs to breach the host cell wall and invade epidermal cells [14]. Insect pests, by contrast, use specialized mouthparts to pierce leaves or stems and feed on plant sap [15]. These diverse modes of attack underscore the central role of early cell‐surface immune recognition in activating rice immunity and preventing pathogen establishment.

#### 2.1.2 PRR‐Mediated Perception of Extracellular Immunecues

PRRs initiate plant immunity by perceiving PAMPs or host‐derived danger signals at the cell surface (Figure 2). A well‐characterized example is OsFLS2, which recognizes flg22, a conserved epitope of bacterial flagellin, and subsequently recruits SERK‐family co‐receptors to form an active immune signaling complex [16, 17, 18]. In rice, OsXA21 perceives the *Xanthomonas oryzae* pv*. Oryzae* ‐secreted sulfated peptide RaxX21, whereas Arabidopsis EFR recognizes the bacterial elongation factor Tu‐derived peptide elf18. These PRRs activate partially overlapping signaling pathways that rely on shared core immune components, including OsSERK2 and OsXB24 [19, 20, 21, 22]. Rice PRRs also recognize conserved microbial cell‐wall components. OsLYP4 and OsLYP6 function as dual receptors for bacterial peptidoglycan and fungal chitin, whereas OsCERK1 and OsCEBiP form a receptor complex that perceives chitin‐derived oligosaccharides [23]. Notably, the recognition capacity of the OsCERK1‐OsCEBiP complex extends beyond fungal pathogens, as it can also detect chitooligosaccharides present in the oral secretions of chewing lepidopteran herbivores [24, 25]. In addition to non‐self‐recognition, PRRs involved in the perception of endogenous danger signals. OsSSR1 and OsBAK1 jointly perceive the apoplastic protein OsSSP1 to enhance fungal resistance [26], while the OsIMP1‐OsRLK5/OsRLK5‐L ligand‐receptor module confers broad‐spectrum pathogen resistance without obvious growth penalties [27]. Moreover, OsCERK1‐OsCEBiP senses rice‐derived damage‐associated molecules, including 31‐β‐D‐cellobiosyl‐glucose and 31‐β‐D‐cellotriosyl‐glucose, which are released from hemicellulose during blast infection [28]. Collectively, PRR‐mediated recognition integrates microbial, herbivore‐derived and host‐derived immune cues, triggering receptor complex assembly, phosphorylation cascades, Ca^2^^+^ influx, ROS production and transcriptional reprogramming to activate downstream defence responses.

**FIGURE 2:**
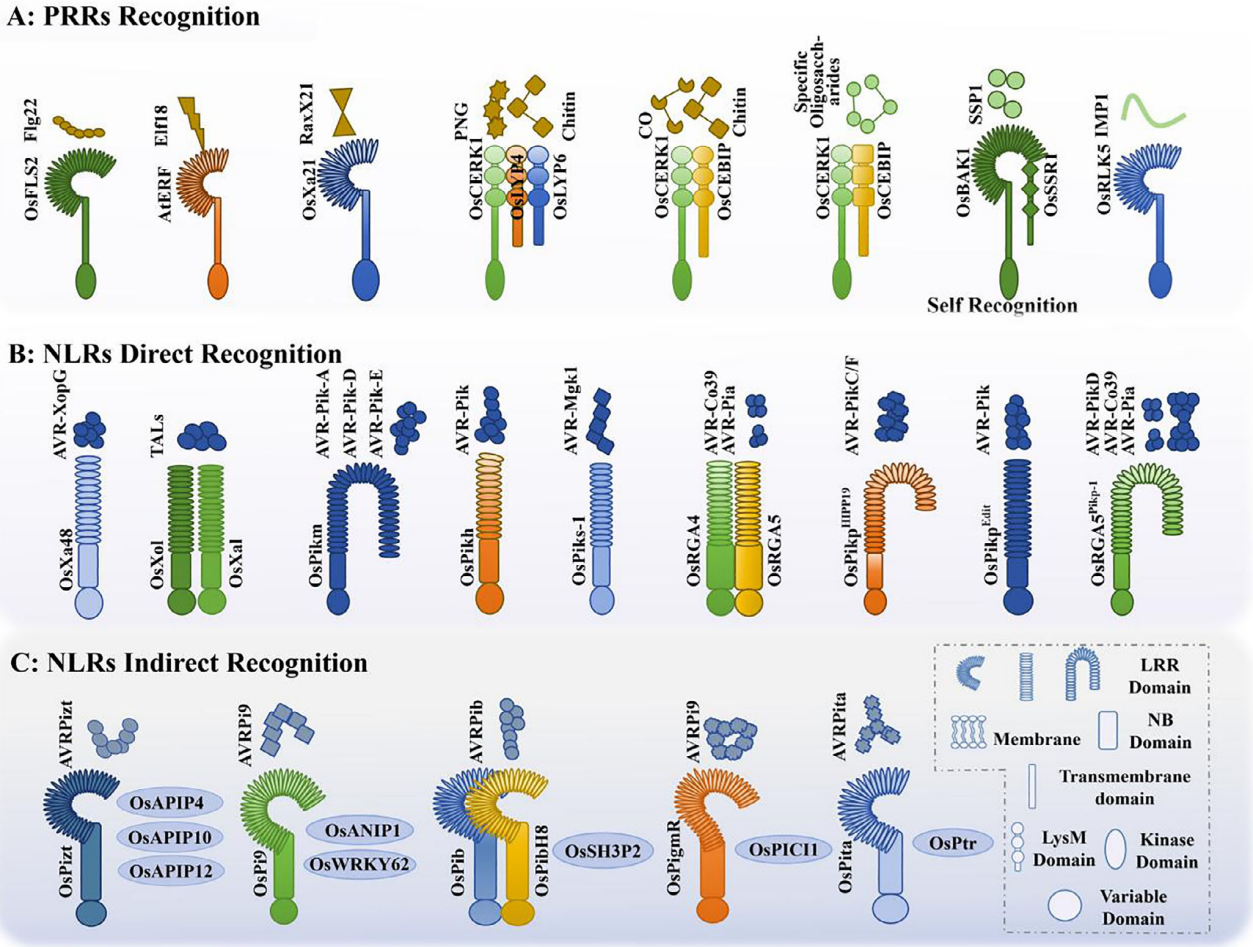
Pathogen recognition by PRRs and NLRs in rice immunity.

PRRs are promising targets for disease‐resistance breeding because they detect conserved pathogen signals early during infection. Their effective deployment, however, requires consideration beyond ligand conservation, including receptor abundance, co‐receptor compatibility, signaling efficiency and genetic‐background effects. Receptor transfer or extracellular‐domain engineering should therefore be validated against diverse pathogen races and across elite varieties. Compared with constitutive activation strategies, breeding designs should perhaps prioritize approaches that maintain low basal signaling while enhancing ligand‐dependent activation, so as to balance disease resistance with growth and yield.

Mechanisms of pathogen recognition by PRRs and NLRs in rice. A: PRRs localized at the cell surface perceive PAMPs or other danger signals. B: NLRs directly recognize pathogen effectors. C: NLRs recognize pathogens indirectly by sensing modifications of host proteins or cellular perturbations caused by pathogen effectors.

### 2.2 Sentinels in the Shadows: NLR Proteins

#### 2.2.1 Overview of NLR Proteins

Nucleotide‐binding leucine‐rich Repeat receptor (NLR) proteins are central components of ETI and represent a major focus of crop immunity research. Most intracellular immune receptors belong to this polymorphic NLR superfamily. Canonical NLR proteins typically contain a C‐terminal leucine‐rich repeat (LRR) domain, a central conserved NB‐ARC domain, and a variable N‐terminal domain. Based on their N‐terminal domains, plant NLRs are generally classified into coiled‐coil NLRs (CNLs), Toll/interleukin‐1 receptor‐like NLRs (TNLs), and RPW8‐like NLRs. Although TNLs are widely distributed in dicotyledonous plants, rice NLRs are predominantly of the CNL type [29]. Genome‐wide analyses have revealed extensive expansion of the NLR repertoire in rice. Following completion of the rice genome sequence, at least 400 NLR‐encoding genes were identified, and subsequent sequencing and annotation of the rice cultivar Tetep revealed 455 NLR genes [30]. Consistent with their functional diversity, NLR proteins display distinct subcellular localizations. Many CNLs and some TNLs are associated with the plasma membrane, whereas other NLRs require nucleocytoplasmic trafficking to execute immune functions [31, 32, 33]. As products of the evolutionary arms race between plants and pathogens, NLRs have diversified to mediate pathogen recognition, immune activation, signal transduction, and downstream defence regulation. However, because NLR‐triggered immunity is often strong and metabolically costly, these receptors are generally maintained in an auto‐inhibited resting state in the absence of pathogen attack [34].

#### 2.2.2 NLR‐Mediated Effector Recognition and Surveillance of Host Targets

A central function of NLR proteins is to activate immune responses upon direct or indirect perception of pathogen effectors or effector‐induced perturbations of host targets. Following failure or suppression of cell‐surface surveillance, NLRs act as intracellular checkpoints that restrict pathogen proliferation. The classical gene‐for‐gene model describes specific correspondence between a host resistance gene and a pathogen a virulence gene, but NLR biology extends beyond simple one‐to‐one recognition. Rice NLRs can bind effectors directly, monitor guarded host proteins, cooperate as sensor‐helper pairs or assemble into resistosome‐like complexes (Figure 2). OsXa48 recognizes XopG, whereas OsXa1 and OsXo1 respond to multiple transcription activator‐like effectors [35, 36, 37]. Integrated‐domain NLRs further diversify recognition: OsPik alleles use heavy‐metal‐associated domains to detect AVR‐Pik variants, and the OsRGA4‐OsRGA5 pair recognizes AVR‐Pia and AVR1‐CO39 [38, 39, 40, 41, 42, 43]. Structure‐guided engineering of OsPikp‐1 and OsRGA5 has broadened effector recognition, demonstrating the potential of rational NLR redesign while highlighting its dependence on pathogen isolate diversity [44, 45, 46].

NLRs can also detect pathogen activity indirectly by monitoring effector‐induced perturbations of host proteins [47]. OsPiz‐t integrates several OsAPIP‐centered surveillance pathways, involving OsAPIP4, OsAPIP10‐OsVOZ1/2 and OsAPIP12, although its relationship with OsAPIP6 remains unresolved [48, 49, 50, 51]. Similarly, AVRPi9 senses disruption of the OsANIP1‐OsWRKY62 complex, OsPib and OsPibH8 are released from OsSH3P2‐mediated repression following AvrPib perception, and OsPigmR protects OsPICI1 from effector‐mediated degradation [52, 53, 54, 55]. OsPi‐ta recognition of AVR‐Pita additionally requires the membrane‐associated protein Ptr [56, 57]. Collectively, direct recognition and host‐target surveillance enable rice NLRs to detect both effector identity and virulence activity, thereby expanding the sensitivity and mechanistic flexibility of ETI.

NLRs can confer strong resistance in rice and are amenable to allele mining, domain engineering and the stacking of sensor–helper receptor modules. Their deployment is nevertheless constrained by pathogen escape, autoactivation and genetic‐background‐dependent fitness costs. Breeding strategies should therefore combine complementary recognition specificities rather than simply increasing NLR copy number, and tune expression levels or promoter activity when constitutive signaling is detrimental. Resistance durability should be evaluated against representative pathogen populations and across developmental stages and environments.

### 2.3 The Frontline: Post‐Recognition Events of PRRs and NLRs

Upon pathogen perception, PRRs rapidly initiate signal transduction, typically by forming immune receptor complexes with appropriate co‐receptors to relay extracellular cues into the cell. These signals are then propagated through multiple downstream pathways, including mitogen‐activated protein kinase (MAPK) cascades, receptor‐like cytoplasmic kinase (RLCK) signaling, calcium (Ca^2^^+^) signaling and phytohormone pathways. Activation of these pathways elicits a range of intracellular immune outputs, such as the rapid burst of ROS and extensive transcriptional reprogramming of defence‐related genes. Similar to PRRs, NLRs also mount rapid responses once pathogen recognition has occurred. In addition to acting as immune signaling modules in their own right, activated NLRs can engage these overlapping downstream pathways to amplify and coordinate immune responses.

#### 2.3.1 MAPK and RLCK Pathways

MAPK signaling constitutes a core module of rice immunity. By relaying pathogen induced signals from cell surface receptors to the nucleus through hierarchical kinase cascades, it activates diverse defence outputs. Importantly, MAPK pathways do not operate in isolation, but are extensively integrated with ROS production, phytoalexin biosynthesis, cell death programmes and hormone signaling, thereby forming a dynamic immune regulatory network. Several MAPK modules that contribute to rice disease resistance have been identified (Figure 3 and Table S2).

**FIGURE 3:**
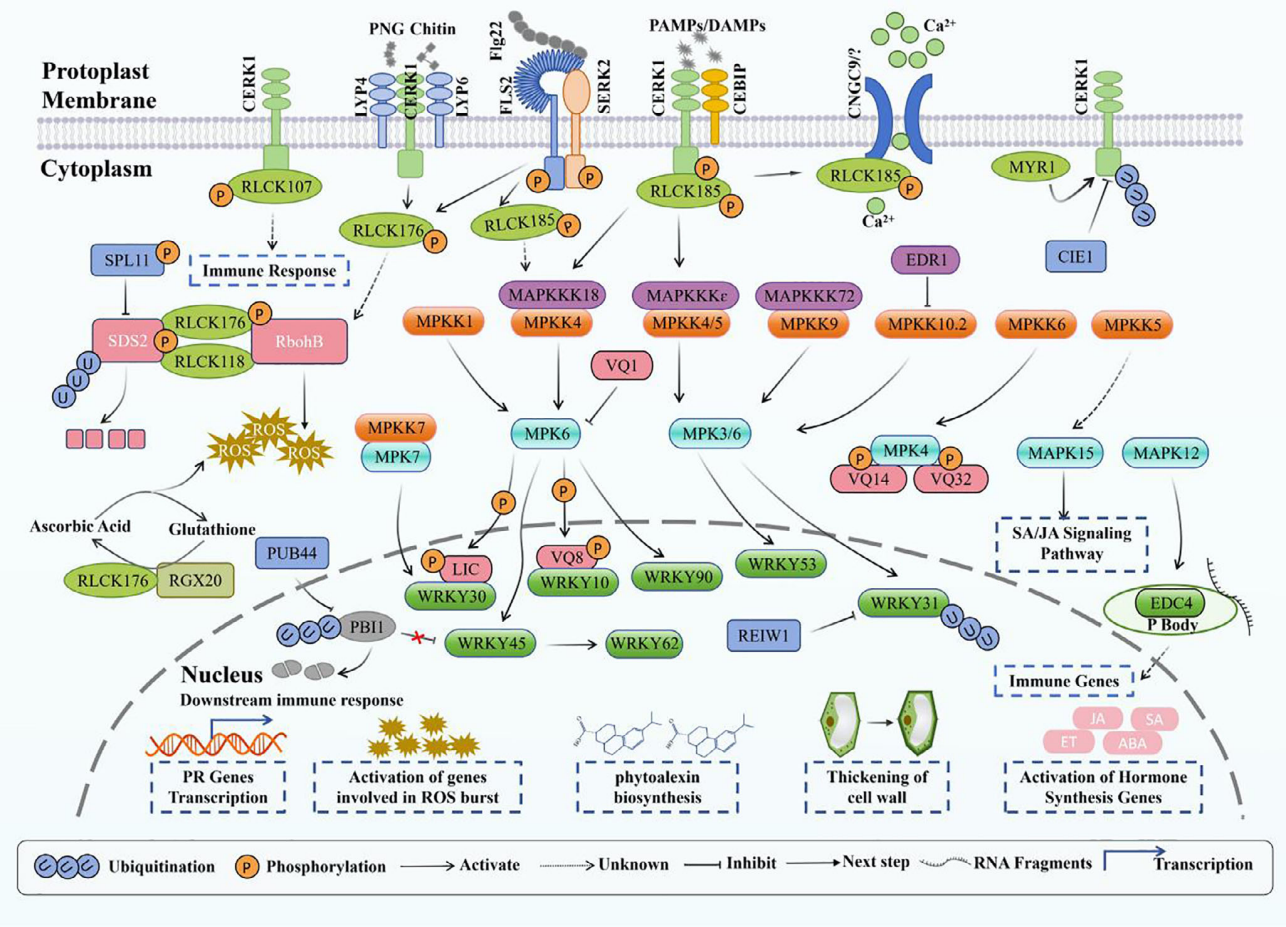
MAPK and RLCK signaling pathways.

OsMPK3 and OsMPK6 are central hubs in the rice immune signaling network, integrating inputs from several MAPK cascades to coordinate transcriptional reprogramming, phytoalexin biosynthesis, and resistance to fungal and bacterial pathogens. Upstream modules involving OsMKK10‐2, OsMKK4, OsMKK1/MEK2, and OsMKK9 converge on OsMPK3/6, which regulate OsWRKY45, OsWRKY90, OsWRKY31, and OsWRKY30, as well as OsVQ8 and OsLIC, thereby linking immune perception to defence‐gene activation and diterpenoid phytoalexin production [58, 59, 60, 61, 62, 63, 64, 65, 67, 68]. Signal strength and duration are further shaped by feedback regulation, protein degradation, and scaffold‐mediated kinase assembly, involving OsEDR1, OsPUB44‐OsPBI1, OsVQ1, OsREIW1, and OsMAPKKK72 [59, 60, 61, 67, 68]. Conversely, OsWRKY53‐mediated inhibition of OsMPK3/6 highlights the importance of constraining MAPK activity to limit defence‐associated costs [69]. Together with the contribution of the OsMPK3‐OsMPK20‐4 interaction to basal defence [66], these findings establish OsMPK3/6 as convergence points at which multiple regulatory mechanisms balance immune robustness with growth and stress adaptation. In addition to the core OsMPK3/6 hub, other MAPK modules further enrich the rice immune signaling network and confer resistance to pathogens. The OsMKK3‐OsMPK7‐OsWRKY30 and OsMKK6‐OsMPK4‐OsVQ14/OsVQ32 cascades positively regulate defence by coordinating pathogenesis‐related responses, cell‐wall maintenance, and metabolic reprogramming [70, 71], whereas OsMPK12 promotes blast resistance through OsEDC4‐dependent immune transcription [73]. By contrast, OsMPK15 negatively regulates resistance to both rice blast and bacterial blight, partly through salicylic acid and jasmonic acid signaling [72]. Thus, the rice MAPK immune network comprises interconnected positive and negative modules that collectively tune response specificity and intensity, thereby enhancing robustness while limiting excessive defence activation.

Because most MAPKs do not directly interact with PRRs, RLCKs act as critical intermediaries that relay immune signals from activated cell‐surface receptors to downstream signaling modules (Figure 3 and Table S2). Beyond linking PRRs to MAPK cascades, RLCKs also regulate rice immunity through MAPK‐independent mechanisms, highlighting that rice immune signaling operates as a multilayered and highly interconnected network rather than a simple linear pathway.

OsCERK1‐containing receptor complexes connect chitin and peptidoglycan perception to OsMPK3/6 activation through OsRLCK185‐OsMAPKKKε/OsMAPKKK18‐OsMKK4/5 signaling modules [74, 75]. Ligand‐specific association of OsCERK1 with OsMYR1, OsLYP4/6, or OsCEBiP, together with regulation by receptor phosphorylation and the E3 ligase OsCIE1, controls kinase activity and recruits downstream OsRLCKs, including OsRLCK107, OsRLCK176, and OsRLCK185 [76, 77, 78, 79]. Similar RLCK modules operate downstream of the OsFLS2‐OsSERK2 complex and also promote OsXa21 expression, indicating that OsRLCKs form shared signaling nodes that connect diverse cell‐surface immune receptors to MAPK activation and reinforce receptor‐mediated immunity [16, 17, 18, 80, 81]. Beyond coupling PRRs to MAPK cascades, RLCKs regulate rice immunity through ROS and hormone signaling. OsRLCK118/176‐OsSDS2 and OsRLCK5‐OsGRX20 modules promote resistance by controlling RbohB‐dependent ROS production and redox homeostasis, with SPL11‐mediated SDS2 degradation providing post‐translational restraint [82, 83]. OsRLCK57, OsRLCK107, and OsRLCK118 also enhance chitin‐ and peptidoglycan‐induced immunity [79], whereas OsNRRB/OsRLCK306 suppresses resistance to bacterial leaf streak through salicylic acid signaling [84]. Thus, RLCKs function as versatile positive and negative regulators that integrate receptor signaling with redox, hormonal, and protein‐turnover pathways.

How NLR‐triggered immunity is connected to MAPK‐ and RLCK‐mediated signaling after effector recognition remains poorly understood, and the mechanisms by which these pathways are activated and coordinated are still largely unresolved. Nevertheless, emerging evidence suggests that ETI can converge with canonical immune signaling modules. For example, OsPi9‐mediated resistance to rice blast involves activation of MAPK signaling together with SA‐ and JA‐dependent defence pathways [85]. In terms of PTI, in addition to the OsCERK1 regulatory module mentioned above, OsMPK17 and OsMKK1 participate in the OsXa21‐mediated resistance pathway against bacterial blight, albeit in distinct ways [86, 87]. Although direct evidence for crosstalk between NLRs, MAPKs and RLCKs in rice remains limited, the extensive overlap between PTI and ETI suggests that these pathways are likely to be deeply interconnected. Defining this cross‐regulatory architecture will therefore be an important direction for future research.

MAPKs and RLCKs occupy highly connected nodes in immune signaling networks and can amplify resistance to diverse pathogens. However, perturbing these central kinases risks pleiotropic effects on development, hormone homeostasis and abiotic stress adaptation; both complete loss of function and constitutive activation may therefore be detrimental. A more practical strategy is to tune signal amplitude and duration by precisely editing negative regulatory motifs, protein‐stability determinants, key phosphorylation sites or inducible cis‐regulatory elements. Candidate alleles should consequently be evaluated not only for endpoint resistance, but also for the controllability of immune dynamics and their yield effects under field conditions both with and without disease pressure.

Following PRR‐mediated perception of pathogen‐associated molecular patterns, RLCKs act as key immune signaling nodes that promote ROS production, Ca^2^^+^ influx and downstream signal relay. RLCK‐dependent activation of MAPK cascades enables signal transmission through MAPKKK‐MAPKK‐MAPK modules to defence‐related genes and regulators, thereby inducing PR gene transcription, phytoalexin biosynthesis, activation of genes involved in ROS burst, thickening of cell wall and activation of hormone synthesis genes. These signaling pathways together link pathogen perception to effective disease resistance in rice.

#### 2.3.2 Small G Protein Activation and Signaling

In parallel with MAPK and RLCK signaling, Rac/Rop‐family small GTPases constitute another important regulatory layer in rice innate immunity. OsRac1 is a representative molecular switch that functions mainly in plasma membrane‐associated immune complexes or microdomains, where it is recruited and activated upon pathogen perception (Figure 4 and Table S3). As a central molecular switch, OsRac1 couples membrane‐proximal signaling to oxidative burst, MAPK activation, and defence amplification. It coordinates OsRbohB‐dependent ROS production and assembles signaling complexes containing OsRACK1A and the OsRAR1‐OsSGT1‐OsHSP90 machinery, while engaging OsMPK3/6‐OsRAI1 signaling and OsAPIP5‐dependent post‐transcriptional regulation [88, 89, 90, 91, 92, 93]. To prevent excessive immune activation, OsRac1 is restrained by OsSPIN6‐mediated GTP hydrolysis‐a process further promoted by OsATG1‐dependent phosphorylation [94, 95]. The related OsRac5‐OsRbohB module similarly enhances blast resistance but is limited by OsATL32‐mediated degradation [96]. Thus, Rac/Rop GTPases integrate ROS and MAPK signaling with multilayered regulatory control, enabling rapid defence activation while constraining the associated cellular costs.

**FIGURE 4:**
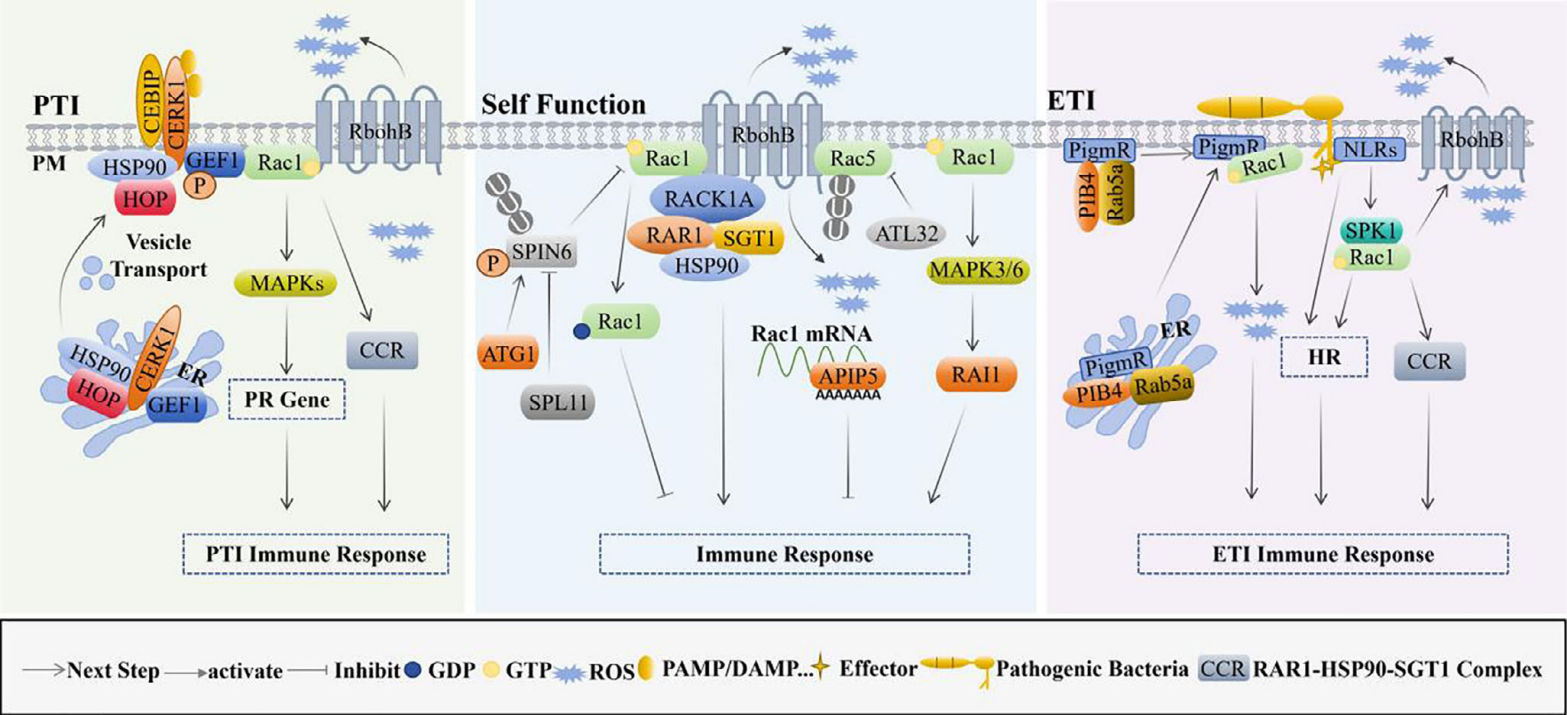
Small G proteins signaling pathway.

As a central hub of immune signaling, OsRac1 occupies a pivotal position in both PTI and ETI. In PRR‐mediated immunity, the OsCERK1‐OsCEBiP receptor complex perceives chitin‐derived non‐self‐signals and transduces them to downstream OsRac1‐dependent signaling pathways, thereby promoting early immune activation. Mechanistically, OsCERK1 phosphorylates OsRacGEF1, a guanine nucleotide exchange factor for OsRac1; the resulting OsCERK1‐OsRacGEF1 complex is then transported from the endoplasmic reticulum to the plasma membrane. Activated OsRacGEF1 subsequently promotes the conversion of OsRac1 into its GTP‐bound active form, enabling rapid initiation of downstream immune responses [97, 98]. Notably, the OsHop/OsSti1a‐OsHsp90 chaperone complex facilitates the maturation and transport of OsCERK1 from the ER to the plasma membrane, and may help coordinate the functional coupling between PRR‐mediated chitin perception and OsRac1‐dependent immune signalling [97, 98].

OsRac1 also functions as a convergence node for NLR‐mediated immunity. OsPit, OsPID3, and OsPigmR engage OsSPK1‐dependent or membrane‐associated mechanisms to activate OsRac1, thereby promoting ROS production, hypersensitive cell death, and blast resistance [99, 100, 101, 102, 103]. Its association with additional NLRs, including OsPib, OsPi9, OsRGA4/OsRGA5, and OsXa1, further suggests that diverse immune receptors share OsRac1‐dependent outputs [99, 100, 101]. These findings, together with its role downstream of PRRs, establish OsRac1 as a mechanistic link between PTI and ETI, through which cell‐surface and intracellular immune receptors converge to coordinate rice defence.

Rac proteins bridge receptor activation to the oxidative burst and hypersensitive cell death, positioning them as key nodes in rice immune signaling. However, constitutively active Rac alleles are unlikely to be agronomically viable because sustained ROS production and cell death can severely impair growth and development. Rather than directly overexpressing active GTPases, more practical strategies could target pathway‐specific guanine nucleotide exchange factors, GTPase‐activating proteins, intracellular trafficking components or infection‐inducible regulators. Such interventions could enhance immunity while minimizing growth penalties. Their agronomic safety should be assessed by monitoring lesion formation, developmental timing, reproductive performance and sensitivity to environmental stresses.

This model summarizes the functions of the small G proteins Rac1 and Rac5 in rice immunity, with Rac1 acting as the central signaling hub. The middle section highlights the intrinsic regulatory features of small G proteins, including their activation from the GDP‐bound inactive state to the GTP‐bound active state, their assembly into immune signaling complexes, and their coordination with other defence pathways. The left section shows their involvement in PRR‐mediated PTI, whereas the right section illustrates their cooperation with NLR proteins in ETI. Together, these activities enable small G proteins to couple pathogen perception with downstream immune signaling and disease‐resistance outputs in rice.

#### 2.3.3 Ca^2^^+^ Signaling Pathway

In eukaryotic cells, Ca^2^^+^ serves not only as an essential mineral nutrient but also as a ubiquitous second messenger. Because excessive cytosolic Ca^2^^+^ is cytotoxic and can interfere with phosphate homeostasis, resting cytosolic Ca^2^^+^ concentrations are maintained at very low levels, typically around 100 nM, far below those in the extracellular space and intracellular Ca^2^^+^ stores. This steep Ca^2^^+^ gradient enables transient changes in cytosolic Ca^2^^+^ concentration to function as rapid and highly sensitive signaling cues [104]. During immune activation, pathogen perception rapidly induces cytosolic Ca^2^^+^ elevations in rice cells. These Ca^2^^+^ signatures are then decoded by Ca^2^^+^ sensors and effectors, which translate spatiotemporal Ca^2^^+^ fluctuations into downstream immune responses. Rice possesses a sophisticated Ca^2^^+^ signaling toolkit, including cyclic nucleotide‐gated channels, calmodulins, calcium‐dependent protein kinases and calcineurin B‐like proteins, which together mediate the generation, transmission, decoding and execution of Ca^2^^+^‐dependent immune signals (Figure 5 and Table S4).

**FIGURE 5:**
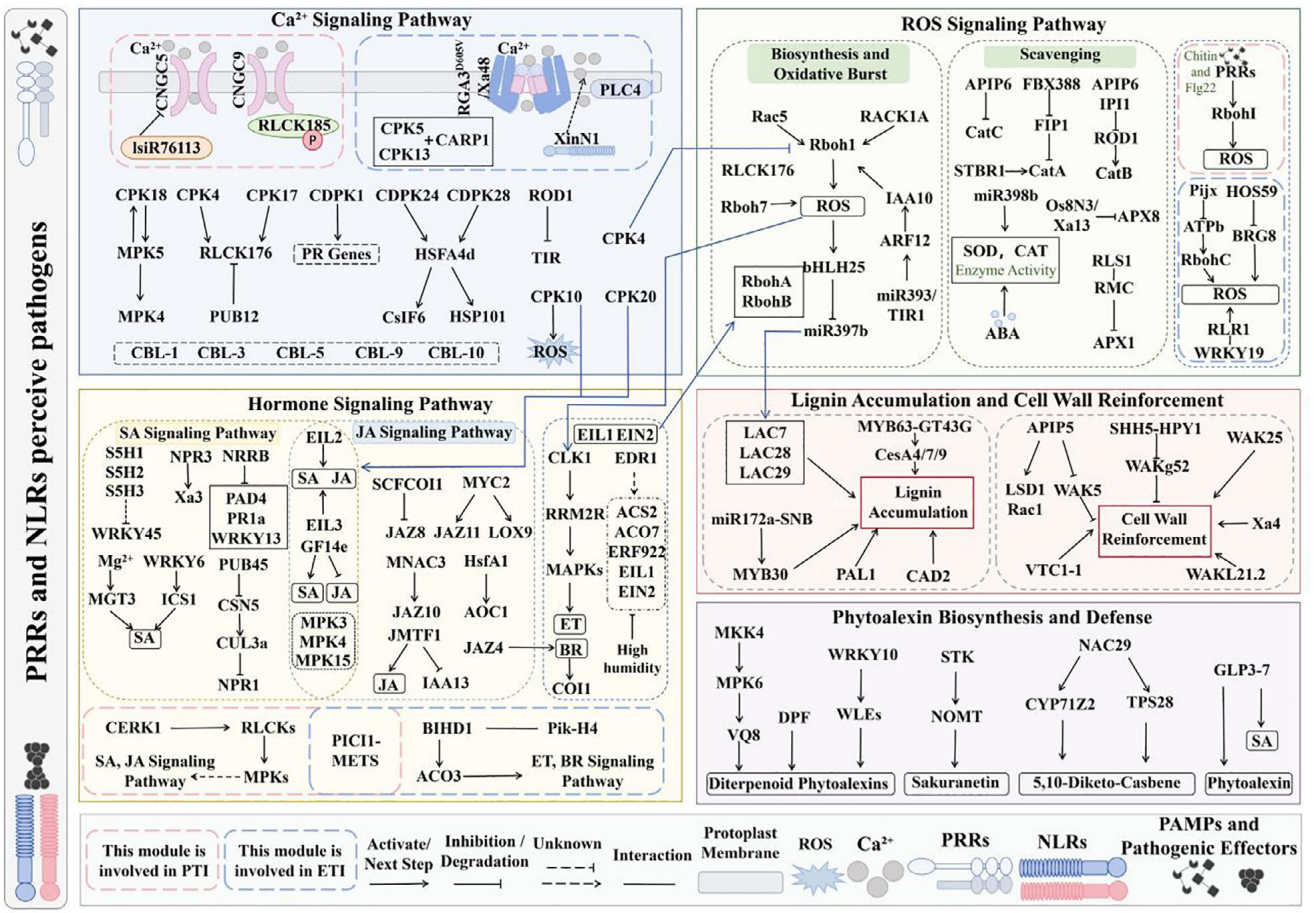
Major signaling pathways involved in rice immune regulation.

Ca^2^^+^ influx provides a shared early signal linking pathogen perception to downstream defence in both PTI and ETI. In PTI, PRR–RLCK modules activate cyclic nucleotide‐gated channels: OsCERK1‐OsRLCK185‐dependent phosphorylation of OsCNGC9 promotes Ca^2^^+^ influx, ROS production and defence‐gene expression, whereas OsCNGC5 supports PAMP responses and blast resistance under negative regulation by OslsiR76113 [105, 106] (Figure 5). In ETI, activated NLRs can directly or indirectly elevate cytosolic Ca^2^^+^. OsRGA3^D605V^ and Xa48 form Ca^2^^+^‐permeable resistosome‐like channels that induce cell death and broad resistance, whereas OsXA21‐induced OsXinN1 also promotes Ca^2^^+^ accumulation through an unresolved mechanism [37, 107, 108] (Figure 5). Thus, PRR‐coupled CNGCs and NLR‐associated channel activities represent complementary routes that converge on Ca^2^^+^ signaling to initiate rice immunity.

Ca^2^^+^‐responsive regulators integrate immune signaling with ROS homeostasis, hormone pathways, transcriptional reprogramming and stress trade‐offs. OsCPK4, OsCPK10 and OsCPK20 modulate Rboh‐dependent ROS dynamics and salicylic acid and jasmonic acid signaling, whereas OsCDPK1 promotes pathogenesis‐related gene expression [111, 112, 113, 114, 115, 116, 117]. By contrast, OsCDPK24/28‐mediated activation of OsHSFA4d prioritizes thermotolerance over immunity, illustrating how Ca^2^^+^ signaling reallocates responses between biotic and abiotic stresses [118]. CBLs are also associated with OsXa21‐mediated resistance, although their mechanistic roles remain unresolved [119]. During ETI, OsPLC4 further couples Ca^2^^+^ and ROS accumulation to lipid peroxidation‐dependent ferroptotic cell death [120]. Thus, Ca^2^^+^ signaling forms an integrative regulatory layer that coordinates defence activation, environmental adaptation and immune‐associated cell death.

Ca^2^^+^ signaling must be tightly constrained to prevent immune activation from compromising growth. The Ca^2^^+^ sensor OsROD1 maintains this balance by limiting TIR‐derived signals and restraining the OsEDS1‐OsPAD4‐OsADR1 complex [121]. More broadly, shared regulators such as OsCPK5 and OsCPK13 position Ca^2^^+^ signaling at the interface of PTI and ETI. Thus, Ca^2^^+^ homeostasis not only limits defence‐associated costs but also enables coordination and mutual reinforcement between the two immune layers.

Ca^2^^+^ channels and sensor kinases rapidly initiate and amplify immune reprogramming. However, because they also regulate rice development and adaptation to heat, drought and salinity, their knockout or constitutive overexpression is likely to cause pleiotropic effects. More promising strategies include allele‐specific or conditional tuning through the engineering of phosphorylation motifs and channel‐gating determinants, or the use of pathogen‐inducible promoters to restrict activation to infection. As Ca^2^^+^ signatures are extensively shared between biotic and abiotic stress responses, candidate alleles should be evaluated under combined pathogen and environmental stresses, rather than against individual pathogens alone, to establish their field efficacy and agronomic safety.

The molecular network controlling rice immunity includes Ca^2^^+^ signaling, phytohormone signaling, reactive oxygen species signaling, as well as late immune responses such as lignin accumulation, cell wall thickening, phytoalexin biosynthesis, and resistance activation. Arrows indicate downstream steps or activation; lines with inhibitory heads, namely T‐shaped lines, indicate inhibition or degradation; dashed lines indicate relationships that remain unclear in immunity; and horizontal lines indicate protein‐protein interactions. Pink dashed boxes denote modules that have been reported in PTI, whereas blue dashed boxes denote modules that have been reported in ETI.

#### 2.3.4 Hormone Signaling Pathway

Phytohormones constitute a major class of small signaling molecules that orchestrate plant immune responses. In rice, a substantial body of evidence has shown that diverse hormones and core components of their signaling pathways collectively underpin a multilayered defence network against pathogen invasion. Although hormone responses generally operate relatively downstream of immune perception, their activation is both rapid and pervasive. Once pathogens are recognized, both PTI and ETI promptly reprogrammed phytohormone signaling to amplify and fine‐tune defence outputs. For instance, upon perception of cognate PAMPs, OsCERK1 phosphorylates OsRLCK185 and activates a MAPK cascade, thereby promoting the biosynthesis of defence associated hormones such as SA and JA [74]. Likewise, the NLR protein OsPik‐H4 interacts with the homeodomain transcription factor OsBIHD1, which directly binds the promoter of the ethylene biosynthetic gene *OsACO3* to modulate the ethylene‐brassinosteroid axis, thereby balancing immune activation with growth [122]. Among the phytohormone pathways implicated in rice immunity, those mediated by SA, JA and ethylene (ET) have been investigated most extensively. These pathways frequently act in synergistic or antagonistic manners, and together determine the amplitude, specificity and outcome of defence responses against distinct pathogen lifestyles (Figure 5 and Table S5).

##### 2.3.4.1 SA and JA Signaling Pathways

SA immunity in rice is coordinated through biosynthesis, turnover and downstream signalling. The PAL pathway appears to provide a major source of SA, linking phenylalanine metabolism and peroxisomal β‐oxidation to SA accumulation and disease resistance [123, 124]. Conversely, S5H‐mediated catabolism constrains SA levels, thereby limiting defence‐gene activation and ROS production under non‐infected conditions [125]. OsNPR1 forms the central signalling hub, whose nuclear accumulation and proteolytic turnover are regulated by redox status and the OsCUL3a‐OsCSN5‐OsPUB45 machinery, whereas OsNPR3‐OsXa5 cooperation further promotes resistance to bacterial blight [126, 127, 128, 129, 130]. SA accumulation is additionally modulated by Mg^2^^+^ homeostasis and transcriptional or RLCK regulators, including OsMGT3, OsNRRB/OsRLCK306 and OsWRKY6 [84, 131, 132]. Thus, rice SA immunity emerges from the integrated control of metabolic flux, signal perception, protein stability and crosstalk with ion and kinase networks.

JA‐mediated immunity in rice is controlled by coordinated regulation of hormone biosynthesis and JAZ repressor turnover. The OsMYC2‐OsLOX9 positive‐feedback loop amplifies JA/JA‐Ile accumulation and blast resistance [133], whereas OsSCF‐OsCOI1‐dependent degradation of JAZ proteins releases defence responses [134]. *OsJAZ4/8/10/11* integrate JA signalling with bacterial, fungal and antiviral immunity, chitin‐triggered PTI and brassinosteroid crosstalk [134, 135, 136, 137]. Environmental signals further reshape this pathway, as the heat‐responsive OsmiR444b.2‐OsHsfA1‐OsAOC1 module enhances JA biosynthesis and blast resistance [138]. Thus, JA functions as an environmentally responsive immune hub that couple metabolic amplification with repressor turnover and hormone crosstalk.

Dynamic crosstalk between SA and JA tailors rice immunity to pathogen lifestyle. SA generally favours resistance to biotrophic and hemibiotrophic pathogens, whereas JA supports defence against necrotrophs, with reciprocal antagonism creating context‐dependent resistance trade‐offs [139]. OsEIL2 and OsEIL3 exemplify this regulatory plasticity by reprogramming SA‐JA balance and generating contrasting resistance outcomes across bacterial, fungal and necrotrophic pathogens [140, 141]. OsGF14e, OsMPK3/4 and OsDR10 further connect kinase and immune‐regulatory networks to distinct SA‐ and JA‐associated outputs [142, 143, 144, 145]. Thus, SA‐JA crosstalk functions as a flexible decision‐making module that aligns defence specificity with pathogen lifestyle while constraining fitness costs.

##### 2.3.4.2 Other Hormone Signaling Pathways

Ethylene (ET) is an important hormonal regulator of rice immunity and is closely connected with both PTI and ETI. The PICI1‐PigmR module promotes ET production by stabilizing methionine synthase, thereby coordinating immune activation; accordingly, exogenous ET treatment or enhanced endogenous ET biosynthesis improves resistance to rice blast and other diseases [55]. ET signaling further promotes blast resistance through OsEIN2 and OsEIL1 by activating ROS production, JA biosynthesis and phytoalexin accumulation [146], while the OsCLK1‐OsRRM2R‐OsMAPKKK18 module enhances resistance by regulating ET biosynthesis [147]. ET‐mediated defence is also shaped by environmental conditions: low humidity favors infection‐induced activation of ET biosynthetic and signaling genes, whereas high humidity suppresses this induction and weakens basal resistance [148]. However, OsEDR1 negatively regulates disease resistance while positively controlling ET biosynthesis, underscoring the context‐dependent and complex role of ET in rice immunity [149].

Beyond SA, JA and ET, additional phytohormones also shape rice immunity. ABA modulates rice black‐streaked dwarf virus (RBSDV) interactions by suppressing JA signaling and altering ROS homeostasis [150]. Hormonal crosstalk further links defence with growth regulation: the JA‐associated transcription factor OsJMTF1 regulates the auxin signaling repressor OsIAA13 to promote bacterial blight resistance [151], while auxin and ROS also contribute to defence against brown planthopper. Strigolactones negatively regulate blast resistance, as reduced strigolactone levels activate JA and sugar signaling and enhance flavonoid phytoalexin accumulation [152]. These studies highlight the broader hormonal network that integrates defence, growth and stress responses in rice immunity.

Hormone pathways offer considerable scope for engineering disease resistance, but their effects depend strongly on pathogen lifestyle and environmental context. Unidirectional enhancement of SA, JA or ethylene signaling can increase resistance to one pathogen class while promoting susceptibility to another or reducing fertility and yield through excessive resource allocation to defence. Hormone engineering should therefore favor context‐dependent regulators, tissue‐specific expression or infection‐inducible promoters over global activation or repression. Candidate strategies should be tested against biotrophic, hemibiotrophic and necrotrophic pathogens, with parallel measurements of hormone levels, defence outputs and yield components. Such multidimensional evaluation is essential to distinguish precise immune tuning from generalized stress activation and to establish agronomic value.

#### 2.3.5 ROS Signaling Pathway

Reactive oxygen species (ROS) are highly reactive oxygen‐containing molecules and free radicals generated during basal metabolism and rapidly accumulated upon stress challenge. In plants, the major ROS species include superoxide anion (O_2_^−^), hydrogen peroxide (H_2_O_2_) and hydroxyl radical (·OH). During immune responses, ROS act not only as antimicrobial agents but also as key signaling molecules that orchestrate defence activation (Figure 5 and Table S6).

Defence‐associated ROS production in rice is dominated by plasma membrane‐localized RBOHs, which integrate inputs from Rac GTPases, RLCKs, CPKs, RACK1A and auxin‐associated signaling. The OsRboh1‐OsRac5, OsRLCK176‐OsRboh7 and OsRACK1A‐Rboh1 modules promote resistance to fungal pathogens, whereas the OsmiR393‐OsTIR1‐OsIAA10‐OsARF12 pathway connects auxin signaling to Rboh‐dependent ROS production [82, 96, 153, 154]. However, OsCPK4‐mediated suppression of Rboh1‐dependent immunity illustrates that RBOH phosphorylation can produce context‐dependent outcomes [113]. Thus, RBOHs function as convergence points that translate diverse immune inputs into spatially and temporally controlled oxidative bursts while limiting ROS‐associated damage.

ROS function as both antimicrobial effectors and signaling molecules in rice immunity. During *M. oryzae* infection, ROS directly damage fungal cellular components and promote hypersensitive cell death, thereby restricting pathogen growth and spread [155, 156, 157]. H_2_O_2_ also reinforces defence through redox‐sensitive regulatory pathways: oxidation of OsbHLH25 promotes OsmiR397b‐dependent lignification, whereas activation of the OsCLK1‐OsRRM2R‐OsRNPS1 module favors production of the immune‐promoting OsMAPKKK18α transcript [147, 158]. Thus, ROS couple direct pathogen restriction and localized cell death with cell‐wall reinforcement and post‐transcriptional immune reprogramming.

Antioxidant enzymes actively shape rice immunity by balancing antimicrobial ROS accumulation against host oxidative damage. Catalase activity is controlled by multilayered regulatory modules: OsAPIP6‐mediated OsCatC degradation, FIP1‐dependent inhibition of OsCatA and OsROD1‐directed recruitment of OsCatB modulate ROS clearance and disease resistance, whereas OsCatA‐OsSTBR1 links redox homeostasis to both immunity and saline–alkali tolerance [159, 160, 161, 162]. SOD and APX pathways similarly integrate hormonal and immune signals. OsmiR398b promotes resistance through SOD‐associated H_2_O_2_ accumulation, whereas ABA‐induced SOD and CAT expression suppresses antiviral defence [150, 163]. The OsRLS1‐OsRMC module and Os8N3/OsXa13 constrain APX‐dependent immunity, whereas OsAPX1 coordinates the early RBOH‐dependent oxidative burst with subsequent ROS detoxification and SA signaling [164, 165, 166]. Thus, ROS‐scavenging enzymes are dynamic immune regulators that determine the timing and magnitude of oxidative defence rather than merely functioning as constitutive detoxification systems.

ROS constitute a shared effector and signalling output of PTI and ETI in rice. In PTI, OsRbohI is the predominant NADPH oxidase responsible for chitin‐ and flg22‐induced oxidative bursts [167]. In ETI, NLRs regulate ROS homeostasis through diverse mechanisms: OsPijx stabilizes OsRbohC indirectly by promoting OsATPb degradation, OsRLR1‐OsWRKY19 signaling controls ROS‐associated defence and cell death, and OsHOS59‐dependent turnover of OsBRG8 restrains HR activation [168, 169, 170]. Thus, PRR‐ and NLR‐mediated immunity converge on tightly regulated ROS production, with oxidase activation and immune‐receptor turnover balancing pathogen restriction against defence‐associated fitness costs.

Effective use of ROS in disease‐resistance breeding requires control over the timing, location and magnitude of the oxidative burst, rather than maximizing ROS production. Early and transient ROS accumulation can restrict pathogen invasion, whereas sustained ROS elevation damages host tissues and may favor necrotrophic pathogens. Practical strategies could therefore tune RBOH regulators, antioxidant enzymes, or their phosphorylation and degradation determinants to adjust the onset and activation threshold of ROS production. Candidate alleles should be evaluated for ROS dynamics, tissue damage and yield performance, particularly under heat, drought and salinity, which perturb redox homeostasis and may alter their field effectiveness.

### 2.4 Erecting Barriers and Deploying Toxins: The Synergy of Lignin Reinforcement and Phytoalexin Defense

#### 2.4.1 Lignin Accumulation and Cell Wall Reinforcement

Upon pathogen attack, the rice cell wall undergoes rapid remodeling, with lignin deposition serving as a key physical barrier against pathogen invasion (Figure 5 and Table S7). Cell‐wall reinforcement is an active immune output coordinated through cellulose and lignin biosynthesis. The OsMYB63‐OsGT43G module, Xa4 and lignin‐associated regulators and enzymes‐including OsmiR172a‐OsSNB, OsTB1, OsPAL1, OsCAD2 and OsLAC7/28/29‐strengthen the wall and restrict bacterial and fungal invasion [158, 171, 172, 173, 174, 175, 176]. Wall‐associated kinases connect these structural changes to immune signaling: OsSHH5‐OsHPY1 and OsAPIP5 suppress WAK‐dependent defence, whereas OsWAKL21.2 and OsWAK25 exert pathogen‐dependent effects [93, 177, 178, 179]. OsVTC1‐1 further links wall composition to hormone signaling [180]. Thus, the cell wall functions as a dynamic immune interface that integrates structural defence with transcriptional, receptor‐mediated and hormonal regulation (Figure 5 and Table S7).

#### 2.4.2 Phytoalexin Biosynthesis and Defense

To resist pathogen invasion, rice produces diverse phytoalexins, a class of secondary metabolites with direct antimicrobial activity. Rice phytoalexins are broadly classified into diterpenoid phytoalexins (DPs) and flavonoid/phenylamide phytoalexins. These compounds contribute to immunity not only by inhibiting pathogen growth but also by modulating immune signaling and cooperating with other defence systems, thereby forming an important layer of the rice immune network [181] (Figure 5 and Table S7). For example, sakuranetin accumulation enhances blast resistance by counteracting pathogen effector‐targeted clathrin‐mediated endocytosis [182]. DP biosynthetic genes are also strongly induced in procambial cells, leading to phytoalexin accumulation in vascular tissues and restricting the spread of *M. oryzae* [183]. In addition, the casbane‐type phytoalexin ent‐10‐oxodepressin confers resistance against fungal blast pathogens in rice [184].

Rice phytoalexin production is coordinated by MAPK signaling and branch‐specific transcriptional regulators. DPF and the OsMKK4‐OsMPK6‐OsVQ8‐OsWRKY10 module activate diterpenoid phytoalexin biosynthetic gene clusters in response to pathogen and abiotic cues [62, 63, 185, 186]. OsGLP3‐7 further links redox activity and JA signaling to phytoalexin metabolism, whereas OsNAC29 and OsSTK selectively promote phytocassane E and sakuranetin biosynthesis, respectively [187, 188, 189]. Thus, rice integrates shared immune signals with pathway‐specific regulators to generate distinct antimicrobial metabolite profiles (Figure 5 and Table S7).

Cell‐wall reinforcement and phytoalexin biosynthesis are terminal immune outputs that offer greater mechanistic precision than engineering upstream signaling hubs and may reduce unintended network‐wide effects. However, redirecting photosynthate and carbon toward lignin or specialized metabolites can alter plant architecture, straw digestibility, grain quality and yield. Promoter editing that enables vascular‐specific or infection‐site‐inducible expression is therefore preferable to constitutive overexpression, allowing defensive products to accumulate where and when they are needed. The most valuable breeding targets will be natural alleles or edited variants that maintain a favorable balance between defence metabolism and agronomic performance under normal field conditions, rather than candidates validated only in greenhouse or artificial‐inoculation assays.

### 2.5 Pathogen Evasion and Retaliation

Pathogens evade cell‐surface immunity by disrupting ligand perception and receptor‐proximal signaling. UvGH18.1 removes immunogenic chitin and prevents OsCEBiP‐OsCERK1 complex formation, facilitating floral invasion by Ustilaginoidea virens [190]. The conserved fungal effector NIS1 instead targets OsBAK1 and OsBIK1, suppressing kinase activity and uncoupling PRR signaling from NADPH oxidase‐dependent ROS production; accordingly, loss of NIS1 reduces *M. oryzae* virulence [191]. These examples illustrate how pathogens converge on early PTI nodes to block both immune recognition and signal amplification.

Pathogen effectors suppress rice immunity by converging on MAPK signaling and ROS homeostasis. AvrPiz‐t and AvrPib disrupt APIP6‐dependent defence and OsMAPKKK72‐OsMKK9 signaling, respectively, whereas SCRE6 alters OsMPK6 phosphorylation and turnover to favor infection [51, 68, 192]. Effectors also redirect ROS dynamics in opposing directions according to pathogen lifestyle: AGLIP1 inhibits OsAPX8 to promote damaging ROS accumulation, whereas UvCBP1 and AvrPiz‐t suppress OsRACK1A‐OsRBOHB‐dependent ROS production or enhance OsCatC‐mediated H_2_O_2_ removal [153, 159, 193]. AvrPiz‐t additionally mimics OsROD1 to hijack Ca^2^^+^‐associated ROS regulation, while a fungal long non‐coding RNA suppresses immunity by sequestering a host miRNA [162, 194].

Beyond PRR and MAPK signaling, pathogen effectors target transcriptional regulation, apoplastic redox balance, defence metabolism and protein turnover. SCRE7 suppresses OsLBD11/12‐dependent immune transcription, MoAo1 disrupts OsAO3/4‐mediated apoplastic ROS regulation, and MoBys1 promotes OsCAD2 degradation to reduce lignin and JA accumulation [174, 195, 196]. Other effectors inhibit key immune regulators: XopP suppresses OsPUB44‐dependent PTI, AvrPi9 destabilizes OsPICI1, SCRE1 inhibits host ATPase activity and Gas2 manipulates OsSnRK1β1A‐associated processes [55, 197, 198, 200]. These interactions can generate reciprocal molecular conflicts, exemplified by mutual destabilization between AvrPi9 and the rice E3 ligase OsRGLG5 [199]. Collectively, pathogen virulence depends on coordinated disruption of multiple host defence layers rather than inhibition of a single immune pathway.

### 2.6 From Back‐to‐Back to Synergy: The Integration of PTI and ETI

Increasing evidence indicates that PTI and ETI are not strictly separated defence layers, but instead share downstream outputs and cooperate to form an integrated immune network. OsPICI1 acts as a central hub in this integration by stabilizing methionine synthase and activating the methionine‐ethylene immune pathway. Although AvrPi9 targets OsPICI1 for degradation to suppress PTI, NLR proteins protect PICI1 from effector‐mediated turnover, thereby restoring this defence cascade [55]. OsCPK5 and OsCPK13 provide another example of PTI‐ETI coupling: they promote early PTI responses while restraining CARP1‐dependent immune activation, whereas effector‐mediated disruption of OsCPK5/13 relieves this repression and triggers ETI [109]. Similarly, OsXinN1 functions downstream of Os*XA21* as part of a PRR‐NLR signaling relay, amplifying PTI outputs and activating ETI without requiring redundant NLR‐mediated pattern recognition [108]. Together, these studies show that rice immunity relies on shared signaling nodes and guarded PTI components to coordinate and amplify PRR‐ and NLR‐mediated defence responses.

### 2.7 The Rice Microbiome as an Extended Layer of Disease Resistance

Rice‐associated microbial communities add an ecological layer to host immunity. Microbiota can provide direct protection before pathogen invasion through resource competition, niche exclusion and antimicrobial production by microorganisms colonizing the rhizosphere, phyllosphere and seed endosphere. For example, phyllosphere‐derived Enterobacter, Pantoea and Pseudomonas strains can directly inhibit Magnaporthe oryzae by producing volatile or non‐volatile antimicrobial compounds. The microbiota can also indirectly shape systemic host immunity. Early colonization by seed endophytes influences resistance‐associated phenotypes, suggesting that vertically transmitted microorganisms can programmed subsequent disease outcomes from the earliest stages of development [201]. Moreover, phyllosphere homeostasis is genetically coupled to host defence metabolism: perturbation of *OsPAL02* and its product 4‐hydroxycinnamic acid disrupts leaf microbial communities and substantially increases disease susceptibility [202]. Together, these findings indicate that disease resistance in rice is an emergent property of interactions among host genotype, resident microbiota and the environment, rather than a trait determined solely by the plant genome. This perspective is shifting resistance breeding from the accumulation of individual resistance genes toward holobiont‐informed crop improvement.

### 2.8 From Immune Mechanisms to Breeding Targets

The studies discussed above reveal a multilayered immune network comprising PRR‐ and NLR‐mediated pathogen recognition and downstream RLCK, MAPK, Ca^2^^+^, ROS, hormone, phytoalexin and cell‐wall‐remodeling pathways. However, mechanistic importance does not necessarily predict breeding value. Many immune regulators also participate in development, metabolic homeostasis or abiotic stress responses, and their manipulation can therefore cause unacceptable pleiotropic effects. Conversely, host susceptibility factors that function primarily during specific infections may have limited roles in core immunity but greater translational potential if their modification imposes little penalty on normal growth. Candidate targets should therefore be prioritized not solely according to resistance strength, but also by considering biological function, risk of pathogen adaptation, dependence on genetic background, baseline fitness costs and field stability (Table S1).

PRRs recognize PAMPs that are often conserved within pathogen groups and are activated mainly upon infection, offering the potential for broad‐spectrum resistance with relatively low basal immune costs. However, their effectiveness depends on compatible co‐receptors and downstream signaling components, which must be considered during receptor transfer or engineering. NLRs generally confer stronger resistance but often recognize a narrower range of effectors and can be defeated by effector variation. Excessive NLR expression can also trigger uncontrolled signaling and autoimmunity. NLR‐based breeding should therefore emphasize the stacking of complementary recognition specificities, allele optimization and expression‐dosage control. RLCKs, MAPKs, Ca^2^^+^, ROS and phytohormones form shared downstream nodes for multiple immune receptors, creating opportunities for broad‐spectrum resistance but also substantial pleiotropic risk. Because these pathways also regulate growth, development and abiotic stress adaptation, complete loss of function or constitutive activation can disrupt immune homeostasis (Table S1). Their rapid, transient and dose‐dependent dynamics further favor precise tuning over binary manipulation. Editing phosphorylation sites, protein‐interaction interfaces and cis‐regulatory elements, or introducing pathogen‐inducible expression, may therefore provide more practical routes to enhance immunity while preserving agronomic performance.

## 3 Genetic Strategies for Coordinating Disease Resistance and Yield in Rice

The preceding sections assessed the breeding potential of PRR‐ and NLR‐mediated recognition and downstream immune components, including RLCKs and MAPKs, thereby identifying intervention points across multiple layers of rice immunity. However, stronger immunity does not necessarily confer greater breeding value. Persistent immune activation can redirect resources from growth and reproduction toward defence, resulting in dwarfism, lesion‐mimic phenotypes, reduced fertility or yield penalties. The central objective of modern resistance breeding is therefore shifting from maximizing immune activity to quantitatively tuning defence in a pathogen‐, tissue‐ and developmental‐stage‐specific manner, thereby jointly optimizing resistance strength, spectrum, durability and yield stability.

Current strategies can be grouped into three broad categories according to the source of resistance and the precision of immune regulation. First, natural variation and induced mutagenesis can identify resistance alleles with minimal or no yield penalties. Second, genome editing and expression engineering can precisely modify susceptibility factors, immune‐regulatory nodes, cis‐regulatory elements or post‐translational modification sites. Third, genetic recombination and marker‐assisted selection can combine resistance genes with complementary recognition spectra or modes of action (Table S1).

### 3.1 Mining Disease‐Resistance Alleles from Untargeted Genetic Variation

Natural variation and mutagenized populations generate alleles spanning complete or partial loss of function, altered protein interactions and quantitative changes in gene expression, providing valuable resources for identifying resistance with low fitness costs. Derived from an EMS‐induced rice mutant population, the broad‐spectrum resistance locus *OsBSR‐K1* confers elevated levels of resistance against both bacterial blight and rice blast. The mutant exhibits enhanced resistance to all tested isolates of both pathogens‐including 10 *Xoo* isolates and 7 *M. oryzae* isolates‐while exerting minimal negative impact on major agronomic traits. Notably, under high rice blast disease pressure, the *bsr‐k1* mutant can partially rescue yield loss compared with the wild type [203]. Similarly, a natural *sbr1* mutant with an insertion disrupting the OsSGR gene increases sheath blight resistance by reducing expression of the CKX gene family and elevating cytokinin content, while maintaining wild‐type agronomic traits including yield [204]. The naturally occurring recessive *rod1* allele identified in the japonica rice TP309 enhances resistance against multiple major diseases‐including blast, bacterial blight, and sheath blight‐and, importantly, limits yield losses under natural disease pressure compared with the wild type, despite showing a slight growth penalty under disease‐free conditions (Figure 6).

**FIGURE 6:**
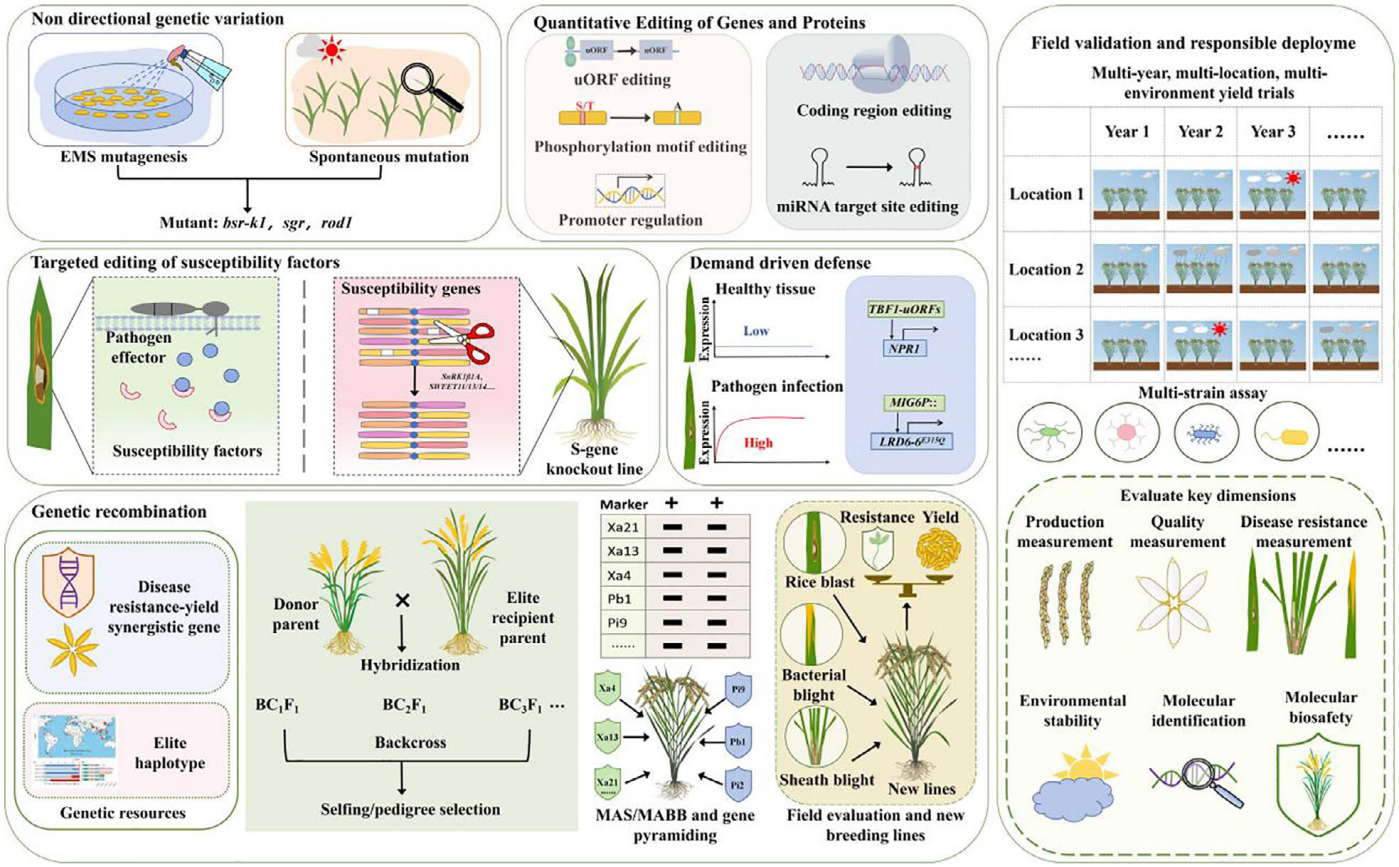
Design strategies for disease‐resistant rice.

The value of untargeted variation lies not only in generating strong resistance phenotypes but also in providing allelic series with different functional strengths. Compared with constitutive activation of NLR‐, ROS‐ or defence hormone‐dependent pathways, moderate alleles are more likely to maintain immune outputs within an effective range without substantially restricting growth, and may therefore offer greater translational potential. Resistance screens should consequently extend beyond lesion area or disease index to include growth, heading date, fertility, yield and grain quality under disease‐free conditions, together with the yield losses prevented under disease pressure. Only alleles that retain stable effects across genetic backgrounds, pathogen populations and field environments should be considered genuinely low‐cost targets for resistance breeding (Table S1).

### 3.2 Precision Engineering of Disease Resistance through Targeted Genetic Variation

Genome editing enables broad‐spectrum or pathogen‐specific resistance to be introduced while minimizing yield penalties. Relevant strategies include inactivating susceptibility factors, uncoupling lesion‐mimic resistance from growth defects and quantitatively tuning genes that jointly influence immunity and yield.

#### 3.2.1 Precision Engineering of Disease Resistance through Susceptibility‐factor Inactivation

Susceptibility factors are host components exploited by pathogens for entry, nutrient acquisition or immune suppression. Disrupting non‐essential susceptibility factors can confer resistance without maintaining constitutively high immune activity and may therefore impose lower growth costs than direct activation of defence pathways. For example, deletion of the long non‐coding RNA RESIS prevents its association with the NatA complex, enhances defence‐associated translation during infection, and confers broad‐spectrum resistance against multiple major diseases‐including bacterial blight, rice blast, and bacterial leaf streak‐without appreciable penalties to yield‐related traits; in fact, resis mutants partially rescue yield loss under disease pressure, whereas direct knockdown of *NatA* complex components causes severe growth defects and yield penalties [205]. Loss of *OsSnRK1β1A* enhances resistance to multiple fungal diseases‐including rice blast, sheath blight, false smut, brown spot, bakanae, and head blight‐without detectable penalties to major agronomic traits; notably, *snrk1β1a* mutants maintained normal growth and yield in field trials across multiple locations under natural disease infection [200]. Loss of *OsRNG1* or *OsRNG3* similarly enhances resistance to both rice blast and bacterial blight without detectable penalties to major agronomic traits under the conditions tested [206]. Targeted disruption of *OsCPR5.1* in the japonica variety Kitaake also confers strong resistance to rice yellow mottle virus (RYMV) while avoiding the severe dwarfism associated with some autoimmunity mutants, as *oscpr5.1* knockout lines showed no substantial growth defects compared with wild type under controlled conditions [207].

However, many susceptibility genes also contribute to transport, metabolism or development, making complete knockout potentially detrimental. In such cases, selectively disrupting pathogen exploitation is preferable to abolishing host‐gene function. Editing TAL‐effector‐binding elements in the promoters of *OsSWEET11*, *OsSWEET13*, and *OsSWEET14* prevents pathogen‐induced transcription while preserving normal physiological expression, conferring resistance to multiple bacterial blight strains [208]. This principle can be extended to other effector‐target systems by editing effector‐binding sites, protein‐interaction interfaces or pathogen‐responsive cis‐regulatory elements (Figure 6).

Multiplex editing can further broaden resistance. Simultaneous modification of *OsPi21*, *OsBsr‐d1* and *OsXa5* generated pbx lines with enhanced resistance to both blast and bacterial blight without significant changes in plant height or thousand‐grain weight [209]. Nevertheless, these traits alone cannot exclude yield penalties. Effective panicle number, grain number, fertility and plot yield must also be assessed, and multiplex lines should be compared with the corresponding single mutants to identify epistatic costs and determine whether gains in resistance breadth justify any agronomic trade‐offs (Figure 6).

#### 3.2.2 Quantitative Editing of Coding Sequences, Cis‐Regulatory Elements and Post‐translational Modification Sites

Complete knockout is rarely optimal for pleiotropic genes that regulate both immunity and development. Editing protein‐interaction interfaces, phosphorylation motifs, miRNA‐binding sites, promoters or upstream open reading frames (uORFs) can instead generate quantitative alleles that preserve basal physiological functions while resetting immune activation thresholds during infection (Figure 6 and Table S1).

Targeted modification of phosphorylation motifs in OsCPK18/4 uncoupled the protein from OsMPK5‐mediated regulation while retaining basal kinase activity, improving both resistance and yield [110]. This finding translates MAPK‐Ca^2^^+^ crosstalk into an actionable design principle: for central immune kinases, editing individual regulatory sites may carry less pleiotropic risk than gene knockout. Similarly, multiplex editing of *OsRBL1* generated the *OsRBL1^Δ12^* allele, which caused only mild lesion mimicry but conferred resistance to multiple isolates or races of blast, bacterial blight and false smut while maintaining relatively stable field yield [210]. Thus, engineering autoimmunity‐associated genes should aim to identify an optimal window between defence enhancement and growth impairment rather than maximize immune activation.

Quantitative manipulation of gene expression offers another route to coordinate resistance and productivity. Overexpression of *OsDes1*, *OsGRF6* or the NLR gene *OsBRW1*, suppression of *OsmiR168*, and reduced *OsCSN5* expression enhanced resistance while maintaining or improving yield‐related traits under the conditions tested [129, 211, 212, 213, 214]. However, the absence of obvious growth defects does not demonstrate that the growth‐defence trade‐off has been eliminated. Because constitutive expression phenotypes can vary across environments, quantitative alleles must be evaluated across disease pressures, developmental stages and field conditions to establish their agronomic robustness.

#### 3.2.3 Pathogen‐Inducible Expression and Demand‐Driven Defence

Constitutive expression of resistance genes can impose substantial fitness costs, particularly when it persistently activates NLR‐, ROS‐, SA‐ or JA‐associated immunity. Pathogen‐inducible promoters and stress‐responsive uORFs offer an alternative by maintaining low immune activity in healthy tissues while enabling rapid defence activation after infection (Figure 6 and Table S1).

The early blast‐responsive *OsMIG6P* promoter enables conditional expression of the lesion‐mimic allele *OsLRD6‐6^E315Q^*, suppressing spontaneous immunity under normal conditions while enhancing resistance to blast, bacterial blight and sheath blight after infection [215]. Translation can also be conditionally regulated. The *OsTBF1* cassette uses two pathogen‐responsive Arabidopsis *TBF1* uORFs to maintain low basal *NPR1* abundance and rapidly increase its translation during infection, thereby broadening resistance in rice while reducing the yield penalties associated with constitutive *NPR1* expression. Editing endogenous promoters, 5′ untranslated regions or uORFs could similarly generate conditional or quantitative resistance alleles without introducing foreign resistance genes, although their specificity and environmental stability require validation [216].

Successful demand‐driven defence depends not merely on pathogen responsiveness but on jointly optimizing basal leakage, induction speed, response amplitude, tissue specificity and duration. Slow activation may permit pathogen establishment, whereas excessive basal expression or prolonged induction can retain growth penalties. The central design objective is therefore coordinated temporal, spatial and quantitative control of immunity, rather than the simple replacement of constitutive promoters with inducible ones.

### 3.3 Introgression and Pyramiding of Elite Resistance Alleles

Genetic recombination, marker‐assisted backcrossing and gene pyramiding enable resistance genes with complementary recognition spectra or mechanisms to be introduced into elite varieties, thereby broadening resistance and potentially improving durability. In BRRI dhan100, pyramiding the blast resistance genes *OsPi9* and *OsPb1* with the bacterial blight resistance genes *OsXa4*, *OsXa13* and *OsXa21* generated lines carrying all five loci, several of which combined enhanced resistance with relatively stable yield traits [217]. Similarly, introgression of *OsXa13*, *OsXa21*, *OsPi2* and *OsPi54* into Basmati rice produced PB1885 and PB1886, which combine resistance to bacterial blight and blast with reduced pesticide requirements [218] (Figure 6).

Introgression of individual elite alleles can also protect yield under specific disease pressures. *OsPijx* enhances blast resistance in japonica rice and provides stronger panicle blast resistance when combined with *OsPigm* or *OsPiz‐t* [168]. The indica‐derived *OsSBRR1‐R* allele reduces yield losses caused by severe sheath blight when introduced into japonica backgrounds [219]. By contrast, *OsIPA1‐1D* illustrates the value of a single pleiotropic regulatory allele: across three years of field trials, it increased yield by 10.1‐13.3% under normal conditions and by 30.7%–48.2% under severe blast pressure [220, 221]. Thus, regulatory alleles connecting development and immunity can sometimes achieve coordinated improvement without multilocus pyramiding.

However, increasing NLR copy number does not necessarily improve resistance and yield. NLR combinations can generate dosage imbalance, incompatible interactions, competition for downstream signaling or autoimmunity, and their fitness effects may vary among genetic backgrounds. Pyramiding should therefore prioritize mechanistically complementary receptors that recognize distinct effectors and are compatible within the target variety. Combining NLRs with PRRs, quantitative resistance loci or susceptibility‐gene edits may provide greater durability by reducing dependence on any single recognition event.

### 3.4 Rapid Breeding for Fast Fixation of Pyramided Resistance

Building on these pyramiding strategies, shortening the interval between the assembly of resistance components and the release of stable cultivars remains a major bottleneck in breeding practice. Accelerated breeding offers a systematic solution. Rapid generation advance combined with marker‐assisted selection enables the targeted fixation of desired gene combinations in early generations, while simultaneously eliminating unwanted donor segments and transgenic or genome‐editing cassettes, thereby compressing the conventional multi‐year process of achieving homozygosity to 2‐3 years [5, 6]. High‐throughput disease screening in early generations can further eliminate lines exhibiting autoimmunity or growth penalties, reducing unproductive trial and error. For suitable germplasm, the integration of genomic selection can further accelerate the pyramiding of resistance‐yield traits and expedite the recovery of the elite genetic background [5, 6]. Importantly, however, the principal value of accelerated breeding lies in expediting early‐stage research and development. It cannot replace replicated, multi‐environment and multi‐year field evaluations against representative pathogen races, which remain essential for confirming the durability of resistance and the agronomic safety of pyramided cultivars.

### 3.5 Field Validation, Biosafety and Responsible Deployment

At the later stages of breeding, evaluation reports for candidate materials should systematically document the pathogen species, isolates or races used for testing; the environmental conditions under which disease pressure was imposed; the genetic background of the recipient line; the developmental stage at sampling or phenotypic assessment; and whether yield was measured under disease pressure, standard field conditions or both. Importantly, the designation of ‘broad‐spectrum resistance’ should be restricted to cases that have been rigorously validated against a defined and representative panel of pathogen lineages. Likewise, ‘yield stability’ should be supported by replicated, multi‐year and multi‐location yield trials, rather than inferred from the stability of proxy traits such as plant height or thousand‐grain weight. Such trials are particularly important for targets involving hormone, reactive oxygen species and Ca^2^^+^ signaling networks, whose phenotypic effects are often strongly influenced by the environment (Figure 6).

The development of genome‐edited materials for practical deployment should also be accompanied by rigorous molecular characterization and regulatory assessment. Candidate lines should be derived from independent transformation or editing events and systematically screened for plausible off‐target mutations and tissue‐culture‐induced somaclonal variation. Where applicable, editing constructs should be removed through segregation in subsequent generations. Multiplex editing and large structural variants warrant particular scrutiny, as unintended alterations may not be adequately detected by conventional amplicon sequencing. Regulatory frameworks for genome‐edited crops differ among countries and regions and continue to evolve [222]. Thus, vector design, material traceability, molecular characterization and data‐reporting standards should be planned early in development according to the requirements of the intended deployment region. Finally, the long‐term sustainability of released cultivars will depend on continued field surveillance of pathogen populations and appropriate resistance‐management strategies to delay resistance breakdown and extend cultivar durability.

Based on the source of resistance and the precision of genetic intervention, current breeding strategies can be broadly classified into three categories: mining natural or induced variation to identify disease‐resistance alleles with minimal or no yield penalties; using genome editing and expression engineering to precisely modify susceptibility factors, immune‐regulatory nodes, cis‐regulatory elements or post‐translational modification sites; and pyramiding resistance genes with complementary recognition spectra or modes of action through genetic recombination and marker‐assisted selection. The resulting breeding materials should be evaluated across multiple years and locations under diverse pathogen pressures, with integrated assessment of yield performance, resistance durability and biosafety.

## 4 Conclusions and Perspectives

During rice domestication and agricultural intensification, pathogen‐driven diseases became increasingly important constraints on food security. Yet mechanistic studies of rice immunity emerged relatively late. The first two waves of the Green Revolution prioritized plant architecture and yield potential through semi‐dwarf breeding and heterosis, respectively, while paying less attention to the complexity of immune networks [223]. The proposed third Green Revolution places disease resistance within a broader agenda of sustainable crop health, marking a transition from maximizing yield potential to safeguarding yield stability. Nevertheless, many components and interactions within rice immune networks remain poorly resolved, limiting the rational design of resistant varieties (Figure 1).

Although numerous immune regulators have been identified, only a small proportion have been translated into breeding practice. A major obstacle is the intrinsic growth‐defence trade‐off: excessive immune activation can impair development and reduce yield [224]. The challenge is therefore not simply to introduce stronger resistance genes, but to deploy them without compromising agronomic performance. Likewise, broad‐spectrum resistance should encompass not only activity against diverse pathogens, but also functional stability across genetic backgrounds, developmental stages and production environments. Resistance‐gene combinations must additionally be evaluated for mechanistic complementarity, synergistic effects and potential fitness penalties.

Climate change further intensifies these challenges because heat, drought and disease increasingly occur in combination. Immune engineering that ignores interactions between biotic and abiotic stress responses may produce varieties that resist pathogens under controlled conditions but perform poorly in extreme environments. Regulatory modules such as OsSGS3‐tasiRNA‐OsARF3 and OsCDPK24/28‐OsHSFA4d illustrate how shared signaling nodes coordinate immunity with environmental adaptation [118, 225]. Future breeding should integrate GWAS, multi‐omics and genome editing to identify and precisely tune genetic combinations that retain resistance under compound stresses. The ultimate objective is climate‐resilient rice in which environmental stress does not erode immunity and disease resistance does not compromise yield.

Germplasm resources are the foundational “chips” of agriculture, and their genetic diversity provides the material basis for improving crop disease resistance. Systematic collection, conservation and evaluation of wild relatives, landraces and elite germplasm are therefore essential for identifying resistance genes and alleles suitable for breeding. During rice domestication and modern breeding, strong selection for yield and agronomic performance has led to the loss of many resistance‐associated genetic resources, leaving some cultivated varieties with limited resistance‐gene diversity and high vulnerability under complex disease pressures. By contrast, wild rice species retain abundant ancestral immune diversity, making them valuable reservoirs for future resistance breeding [226]. Genome editing provides a new strategy for directly converting wild rice into crop types better adapted to modern agriculture. In particular, resistance‐gene‐based de novo domestication can preserve the intrinsic resistance repertoire of wild rice while editing domestication‐ and yield‐related loci to improve undesirable traits such as seed shattering, plant height, awn length and panicle architecture. This approach can compress a domestication process that traditionally required thousands of years into only a few years. For example, CRISPR‐Cas9‐mediated editing of seven key genes in wild rice improved plant architecture, awn length, hull color and seed shattering while retaining part of the wild resistance‐gene repertoire [227]. Similarly, genome editing combined with multi‐omics analyses enabled the rapid domestication of the South American allotetraploid wild rice *Oryza* alta into an agriculturally valuable crop type [228]. These advances indicate that wild rice improvement and utilization will become an important frontier for future disease‐resistance breeding. In addition to natural genetic resources, mutagenesis remains a powerful strategy for generating genetic diversity at scale. Mutagenesis‐derived alleles can be directly used for crop improvement and may create traits that are absent from natural populations or were lost during domestication and evolution. With advances in genomics and biotechnology, mutagenesis has evolved from conventional physical and chemical methods into a diversified high‐throughput framework that integrates space‐environment mutagenesis, molecular biology and precise genome editing [229, 230] (Figure 1).

With rapid technological advances, rice breeding is shifting from experience‐driven selection to precision design. High‐throughput sequencing, GWAS and population‐genetic approaches now enable precise mapping and cloning of resistance loci, including rare allelic variants that were previously difficult to detect (Figure 1). In parallel, marker‐assisted selection, gene pyramiding, genome editing and genomic selection allow resistance genes to be efficiently introduced or optimized in elite cultivars while minimizing adverse effects on yield and grain quality. These technologies can shorten breeding cycles, improve selection efficiency and support the coordinated improvement of disease resistance, yield and quality, thereby moving rice breeding toward demand‐oriented design and precision creation. Artificial intelligence and phenomics are further reshaping disease‐resistance breeding. AI‐assisted models can integrate genome‐wide datasets to predict genotype–phenotype relationships and identify superior breeding materials with improved resistance potential. Meanwhile, high‐throughput phenotyping platforms, including unmanned aerial vehicles, Internet‐of‐Things sensors and field robots, can collect images, spectral data and dynamic growth parameters after pathogen infection. Combined with computer vision and machine‐learning algorithms, these platforms enable automatic extraction of disease‐related traits and precise monitoring of large breeding populations. This approach can complement or partially replace manual field evaluation, reducing subjectivity and improving the objectivity, efficiency and predictive power of breeding decisions.

Green and sustainable agricultural development remains a long‐term and far‐reaching objective. Current production models that pursue high yield at the expense of excessive natural‐resource consumption and ecological degradation are ultimately unsustainable. Therefore, the in‐depth discovery and efficient utilization of rice disease‐resistance gene resources, together with their accelerated translation into breeding practice, are of critical importance for ensuring the stable and sustainable production of rice. More broadly, these efforts will help safeguard the ecological security of farmland, promote the transition of agricultural production toward environmentally friendly systems and support a more balanced coexistence between human society and nature.

## Gene Nomenclature Note

All gene symbols are presented in abbreviated form; their full names are listed in Table S1.

## Author Contributions

Deyong Ren and Qian Qian conceived the project. Xinyue Hou, Yuanjiang Cui, and Deyong Ren wrote the manuscript. Deyong Ren and Xinyue Hou edited the manuscript. Chaoqing Ding and Xinyue Hou collected the references and prepared the figures and tables. Deyong Ren and Qian Qian agree to serve as the authors responsible for contact and ensuring communication.

## Conflicts of Interest

The authors declare no conflicts of interest.

## Supporting information




**Supporting File 1**: advs77719‐sup‐0001‐Tables.docx.

**Supporting File 2**: advs77719‐sup‐0002‐TableS1.xls.

## Data Availability

The data that support the findings of this study are available from the corresponding author upon reasonable request.
