# Supplementary material for: Molecular Bases and Genetic Design of Rice Disease Resistance for Optimized Yield and Sustainable Agriculture
Source: Adv Sci (Weinh). 2026 Sep 16;13(58):e77719. doi: 10.1002/advs.77719 (PMC13583107; doi:10.1002/advs.77719)
Supplement: Supplementary file 1 [file ADVS-13-e77719-s002.docx]

Table 1. Major applied strategies for enhancing disease resistance in rice breeding

| **Breeding Strategy** | **Design Principles and Applicable Targets** | **Feasibility and Expected Durability Analysis** | **Major Risks and Impacts** | **Degree of Recommendation** |
| --- | --- | --- | --- | --- |
| Expansion of PRR/NLR recognition spectra and receptor engineering | Expanding recognition spectra against conserved PAMPs or effectors through receptor transfer, site-directed editing, allele mining or structure-guided binding-interface engineering. | Receptor engineering is at early-to-mid-stage development. The more conserved the target ligand/effector and the more complementary the receptor combinations, the higher the theoretical durability; NLR single-gene resistance is generally more vulnerable to pathogen variation. | Incomplete basal signalling and pathway compatibility, as well as pathogen masking or loss of ligands, may limit efficacy. Additional risks include race specificity, pathogen escape, autoimmunity and genetic-background-dependent fitness penalties. | Medium |
| Core signalling engineering involving MAPK, RLCK, small G proteins, phytohormone signalling and ROS | Fine-tuning the innate homeostasis between immunity and growth in a spatiotemporally precise "elastic-band" manner, achieving coordinated improvement of resistance and yield by locally activating defence while globally maintaining photosynthesis. | Broad effects may be achieved, but predictability is low owing to the highly interconnected nature of these nodes. | Developmental defects, redox damage, altered susceptibility to pathogens with different lifestyles, and abiotic-stress trade-offs. | Low |
| Mining resistance alleles from natural variation and mutagenized populations | Screening for moderate-effect, partial-loss-of-function or expression-altered alleles from landraces, wild rice, natural populations and physical/chemical mutagenized populations; prioritizing materials with low growth and yield penalties under disease-free conditions. | Technical path is mature and readily integrated with conventional crossing, backcrossing and marker-assisted selection; durability depends on resistance mechanism, pathogen population structure and genetic background. | Mutagenized populations may carry linked background mutations; phenotypic gains may be background- or environment-specific. Causal relationships need to be confirmed by fine mapping, complementation and/or recapitulation through editing, with evaluation of heading date, seed-setting rate, yield and quality. | High |
| Precision editing of susceptibility genes or effector-binding elements | Prioritizing disruption of pathogen-specific exploitation processes rather than complete ablation of the host gene's physiological function; applicable to non-essential susceptibility factors, effector-binding sites, protein–protein interaction interfaces and pathogen-inducible cis-elements. | CRISPR-based feasibility is high; expected durability is relatively high if the pathogen's dependence on the target host factor is irreplaceable and the host basal function is preserved. | Complete knockout may cause pleiotropic effects, and pathogens may exploit alternative targets or effectors. | Medium |
| Modulation of promoters, cis-elements, uORFs or post-translational modification motifs | "Tuning the intensity" rather than simply knocking out pleiotropic genes that have both immune and developmental functions; editing phosphorylation sites, protein–protein interaction interfaces, promoters, miRNA-binding sites or uORFs to reset immune thresholds. | Enables quantitative or infection-responsive control, suitable for reducing basal defence penalties. | Optimal effect window may be narrow and influenced by genetic background, temperature and abiotic stresses. | Medium |
| Pathogen-inducible and on-demand defence engineering | Maintaining low-level defence in healthy tissues while rapidly upregulating transcription or translation of resistance genes upon infection; focusing on optimizing basal leakage, induction speed, amplitude, tissue specificity and duration. | Proof-of-concept is well established, but breeding applications remain relatively early; if induction is specific and stable, holds promise for combining broad-spectrum resistance with low basal penalties. | Potential issues include leakage expression, insufficient or too-slow induction, and non-specific activation by wounding, drought or temperature. | Medium |
| Gene pyramiding through introgression of resistance genes and mechanism-based complementation | Introducing genes that recognize different effectors or target different pathogens into elite recipients through crossing, backcrossing and marker-assisted selection; pyramiding should emphasize mechanistic complementarity and recipient-background compatibility rather than simply increasing NLR number. | Most mature application in conventional breeding; expected durability is moderate to high when gene recognition spectra are complementary and expression is coordinated in the target background. | Linkage drag, antagonistic epistasis, expression imbalance and breakdown as pathogen populations shift. | High |
| Genomic prediction, phenomics and AI-assisted design | Enables high-throughput screening and prioritization of combinations when large and representative datasets are available. | Maturity for supporting breeding decisions is rapidly increasing, but cross-environment generalizability remains limited; should serve as a complement to genetic and field validation rather than a substitute for pathogen inoculation and yield trials. | Data bias, limited interpretability and poor extrapolation to scenarios outside the training environment. | High |

Table 2. Genes involved in the MAPK and RLCK pathways

| **Gene name** | **Accession Number** | **Protein Category** | **Disease Resistance** | **References** |
| --- | --- | --- | --- | --- |
| MPK3 | Os03g0285800 | Mitogen-activated protein kinase | *M. Oryzae and Xoo* | [58][59][66] |
| MAPK6 | Os06g0154500 | Mitogen-activated protein kinase | *M. Oryzae, Xoo and Xoc* | [58][59][61] |
| MPK20-4 | Os01g0665200 | Mitogen-activated protein kinase | *M. Oryzae and Xoo* | [66] |
| MPK4 | Os10g0533600 | Mitogen-activated protein kinase | *Xoo* | [71] |
| MPK7 | Os06g0699400 | Mitogen-activated protein kinase | *Xoo* | [70] |
| MPK15 | Os11g0271100 | Mitogen-activated protein kinase | *M. Oryzae and Xoo* | [72] |
| MPK12 | Os06g0708000 | Mitogen-activated protein kinase | *M. Oryzae* | [73] |
| MPKK10.2 | Os03g0225100 | Mitogen-activated protein kinase kinase | *M. Oryzae, Xoo and Xoc* | [58][59] |
| MKK4 | Os02g0787300 | Mitogen-activated protein kinase kinase | *M. Oryzae, Xoo and Xoc* | [74][75] |
| MKK1 | Os06g0147800 | Mitogen-activated protein kinase kinase | *M. Oryzae* | [65] |
| MKK9(MPKK5) | Os06g0191500 | Mitogen-activated protein kinase kinase | *M. Oryzae and Xoo* | [68][72] |
| MKK3 | Os06g0473200 | Mitogen-activated protein kinase kinase | *Xoo* | [70] |
| MPKK6 | Os01g0510100 | Mitogen-activated protein kinase kinase | *Xoo* | [71] |
| EDR1 | Os03g0160100 | Mitogen-activated protein kinase kinase kinase | *M. Oryzae, Xoo and Xoc* | [58][59] |
| MAPKKK72 | Os01g0748600 | Mitogen-activated protein kinase kinase kinase | *M. Oryzae* | [68] |
| MAPKKKε | Os04g0660500 | Mitogen-activated protein kinase kinase kinase | *M. Oryzae* | [74] |
| MAPKKK18 | Os03g0764300 | Mitogen-activated protein kinase kinase kinase | *M. Oryzae* | [75] |
| RLCK185 | Os05g0372100 | Receptor like cytoplasmic kinase | *M. Oryzae and Xoo* | [74][75][105][122] |
| RLCK176 | Os05g0110900 | Receptor like cytoplasmic kinase | *M. Oryzae Xoo and Xoc* | [78][82] |
| RLCK107 | Os03g0274800 | Receptor like cytoplasmic kinase | *Xoo* | [79] |
| RLCK57 | Os01g0973500 | Receptor like cytoplasmic kinase | *Xoo* | [80] |
| RLCK102 | Os03g0170400 | Receptor like cytoplasmic kinase | *Xoo* | [80] |
| RLCK118 | Os03g0821900 | Receptor like cytoplasmic kinase | *M. Oryzae and Xoo* | [80] |
| RLCK5 | Os01g0114100 | Receptor like cytoplasmic kinase | *R. solani* | [83] |
| RLCK306 | Os10g0548700 | Receptor like cytoplasmic kinase | *Xoc* | [84] |

Table3. Genes involved in the small G protein pathway

| **Gene name** | **Accession Number** | **Protein Category** | **Disease Resistance** | **References** |
| --- | --- | --- | --- | --- |
| Rac1 | Os01g0229400 | Small molecule GTPase | *M. Oryzae and Xoo* | [88][89] |
| RacGEF1 | Os09g0544800 | Rac1 guanine nucleotide exchange factor | *M. Oryzae* | [95][98] |
| Rac5 | Os02g0834000 | Small molecule GTPase | *M. Oryzae and Xoo* | [96] |
| RACK1A | Os01g0686800 | Activated protein kinase C receptor | *M. Oryzae and Xoo* | [90][91] |
| RAR1 | Os02g0535400 | Innate immune response protein | *M. Oryzae and Xoo* | [90][91] |
| SGT1 | Os01g0624500 | SCF ubiquitin ligase complex subunit | *M. Oryzae and Xoo* | [90][91] |
| HSP90 | Os06g0716700 | Heat shock protein | *M. Oryzae and Xoo* | [90][91] |
| RAI1 | Os03g0135700 | Basic helix loop helix transcription factor | *M. Oryzae* | [92] |
| SPIN6 | Os07g0658300 | GTPase activating protein | *M. Oryzae and Xoo* | [94][95] |
| ATL32 | Os02g0686100 | E3 ubiquitin ligase | *M. Oryzae and Xoo* | [96] |

Table4. Genes involved in the calcium ion pathway

| **Gene name** | **Accession Number** | **Protein Category** | **Disease Resistance** | **References** |
| --- | --- | --- | --- | --- |
| CNGC9 | Os09g0558300 | Cyclic nucleotide gated ion channel | *M. Oryzae* | [105] |
| CNGC5 | Os12g0468500 | Cyclic nucleotide gated ion channel | *M. Oryzae* | [106] |
| RGA3 | Os11g0224900 | NLR protein | *M. Oryzae and Xoo* | [107] |
| Xa48 | Os03g0689866 | NLR protein | *Xoo* | [37] |
| CPK5 | Os02g0685900 | Calcium dependent protein kinase | *M. Oryzae* | [109] |
| CPK13 | Os04g0584600 | Calcium dependent protein kinase | *M. Oryzae* | [109] |
| CPK18 | Os07g0409900 | Calcium dependent protein kinase | *M. Oryzae* | [110] |
| CPK4 | Os02g0126400 | Calcium dependent protein kinase | *M. Oryzae and Xoo* | [111] |
| CPK17 | Os07g0161600 | Calcium dependent protein kinase | *Xoo and Xoc* | [112] |
| CPK10 | Os03g0788500 | Calcium dependent protein kinase | *M. Oryzae* | [114][116] |
| CPK20 | Os07g0568600 | Calcium dependent protein kinase | *M. Oryzae* | [115] |
| CDPK1 | Os03g0128700 | Calcium dependent protein kinase | *M. Oryzae and Xoo* | [117] |
| CDPK24 | Os11g0171500 | Calcium dependent protein kinase | *M. Oryzae Xoo and R. Solani* | [118] |
| CDPK28 | Os12g0169800 | Calcium dependent protein kinase | *M. Oryzae Xoo and R. Solani* | [118] |
| CBL-1 | Os10g0564800 | Calcineurin B-like protein | *Xoo* | [119] |
| CBL-3 | Os03g0626800 | Calcineurin B-like protein | *Xoo* | [119] |
| CBL-5 | Os01g0598200 | Calcineurin B-like protein | *Xoo* | [119] |
| CBL-9 | Os01g0579600 | Calcineurin B-like protein | *Xoo* | [119] |
| CBL-10 | Os01g0711500 | Calcineurin B-like protein | *Xoo* | [119] |
| PLC4 | Os05g0127200 | Phosphatidylinositol specific phosphatase C | *M. Oryzae* | [120] |
| ROD1 | Os06g0128800 | Ca^² +^ sensor | *M. Oryzae Xoo and R. Solani* | [121] |

Table 5. Genes involved in phytohormone signalling pathways

| **Gene name** | **Accession Number** | **Protein Category** | **Disease Resistance** | **References** |
| --- | --- | --- | --- | --- |
| *S5H1* | Os03g0122300 | Flavanone 3-hydroxylase | *M. Oryzae and Xoo* | [125] |
| *S5H2* | Os04g0581000 | Flavanone 3-hydroxylase | *M. Oryzae and Xoo* | [125] |
| *S5H3* | Os10g0536400 | Flavanone 3-hydroxylase | *M. Oryzae and Xoo* | [125] |
| NPR1 | Os01g0194300 | Salicylic acid receptor | *M. Oryzae* and *Xoo* | [126][127][128] |
| CUL3a | Os02g0746000 | Cullin protein | *M. Oryzae* and *Xoo* | [129] |
| CSN5 | Os04g0654700 | COP9 signalosome subunit | *M. Oryzae* and *Xoo* | [129] |
| PUB45 | Os02g0540700 | E3 ubiquitin ligase | *M. Oryzae* and *Xoo* | [129] |
| NPR3 | Os03g0667100 | Salicylic acid receptor | *M. Oryzae* and *Xoo* | [130] |
| MGT3 | Os03g0684400 | Mg^2+^ transporter | *M. Oryzae* and *Xoo* | [131] |
| ICS1 | Os09g0361500 | Isochorismate synthase | *M. Oryzae* | [132] |
| JMTF1 | Os01g0285300 | MYB transcription factor | *Xoo* | [151] |
| LOX9 | Os08g0508800 | Lipoxygenase | *M. Oryzae* | [133] |
| MYC2 | Os10g0575000 | Basic helix loop helix transcription factor | *M. Oryzae* and *Xoo* | [133] |
| JAZ4 | Os09g0401300 | Jasmonic acid Zim domain protein | *RBSDV* | [135] |
| JAZ10 | Os03g0181100 | Jasmonic acid Zim domain protein | *M. Oryzae* | [136][137] |
| JAZ11 | Os03g0180900 | Jasmonic acid Zim domain protein | *M. Oryzae* | [136][137] |
| MNAC3 | Os07g0683200 | NAC transcription factor | *M. Oryzae* and *Xoo* | [136] |
| AOC1 | Os03g0438100 | Allene oxide cyclase | *M. Oryzae* | [138] |
| HsfA1 | Os03g0854500 | Heat shock transcriptional factor | *M. Oryzae* | [138] |
| EIL2 | Os07g0685700 | Ethylene signaling regulators | *M. Oryzae Xoo and R. Solani* | [140] |
| EIL3 | Os09g0490200 | Ethylene signaling regulators | *M. Oryzae Xoo and R. Solani* | [141] |
| GF14e | Os02g0580300 | 14-3-3 protein | *M. Oryzae* and *Xoo* | [142] |
| PICI1 | Os10g0533900 | Deubiquitinase | *M. Oryzae* and *Xoo* | [55] |
| DR10 | Os08g0155900 | Defense-responsive gene | *Xoo* | [145] |
| EIN2 | Os07g0155600 | Ethylene signaling regulators | *M. Oryzae* | [146] |
| EIL1 | Os03g0324200 | Ethylene signaling regulators | *M. Oryzae* | [146] |
| ERF922 | Os01g0752500 | ERF transcription factor | *M. Oryzae* | [148] |
| ACS2 | Os04g0578000 | Aminocyclopropane-1-carboxylate synthase | *M. Oryzae* | [148] |
| ACO7 | Os01g0580500 | Aminocyclopropane-1-carboxylate oxidase | *M. Oryzae* | [148] |
| CLK1 | Os01g0837900 | Protein kinase | *M. Oryzae* | [147] |
| RRM2R | Os06g0566100 | RNA-binding protein | *M. Oryzae* | [147] |

Table 6. Genes involved in the reactive oxygen species pathway

| **Gene name** | **Accession Number** | **Protein Category** | **Disease Resistance** | **References** |
| --- | --- | --- | --- | --- |
| Rboh1 | Os01g0360200 | NADPH oxidase | *M. Oryzae and Xoo* | [113] |
| Rboh7 | Os09g0438000 | NADPH oxidase | *M. Oryzae and Xoo* | [82] |
| bHLH25 | Os01g0196300 | bHLH transcription factor | *M. Oryzae Xoo and R. Solani* | [158] |
| CatC | Os03g0131200 | Catalase | *M. Oryzae* | [159] |
| CatA | Os02g0115700 | Catalase | *M. Oryzae* | [160][161] |
| CatB | Os06g0727200 | Catalase | *M. Oryzae Xoo and R. Solani* | [162] |
| STBR1 | Os08g0562500 | BAHD acyltransferase-like protein | *M. Oryzae* | [161] |
| RLS1 | Os02g0203500 | NB-ARM protein | *Xoo* | [164] |
| RMC | Os04g0659300 | Root meander curling | *Xoo* | [164] |
| APX8 | Os02g0553200 | Chloroplast ascorbate peroxidase | *Xoo* | [165] |
| APX1 | Os03g0285700 | Chloroplast ascorbate peroxidase | *Xoo* | [166] |
| RbohI | Os11g0537400 | NADPH oxidase | *M. Oryzae* | [167] |
| Pijx | Os12g0564800 | NLR protein | *M. Oryzae* | [168] |
| ATPb | Os05g0553000 | ATPase β subunit | *M. Oryzae* | [168] |
| RbohC | Os05g0528000 | NADPH oxidase | *M. Oryzae* | [168] |
| RLR1 | Os10g0163040 | NLR protein | *M. Oryzae* and *Xoo* | [169] |
| BRG8 | Os08g0190300 | NLR protein | *M. Oryzae* and *Xoo* | [170] |
| HOS59 | Os06g0646600 | NLR immune regulator | *M. Oryzae* and *Xoo* | [170] |

Table 7. Participate in the genes of plant protection hormones and cell wall pathways

| **Gene name** | **Accession Number** | **Protein Category** | **Disease Resistance** | **References** |
| --- | --- | --- | --- | --- |
| MYB63 | Os04g0594100 | MYB family transcription factor | *Xoo* | [171] |
| GT43G | Os10g0205300 | Glycosyltransferase 43G | *Xoo* | [171] |
| CesA4 | Os01g0750300 | Cellulose synthase catalytic subunit | *Xoo* | [171] |
| CesA7 | Os10g0467800 | Cellulose synthase catalytic subunit | *Xoo* | [171] |
| CesA9 | Os09g0422500 | Cellulose synthase catalytic subunit | *Xoo* | [171] |
| MYB30 | Os02g0624300 | MYB family transcription factor | *M. Oryzae Xoo and R. Solani* | [173] |
| SNB | Os07g0235800 | APETALA2-like transcription factor | *M. Oryzae Xoo and R. Solani* | [173] |
| TB1 | Os03g0706500 | TCP family transcription factor | *M. Oryzae* | [174] |
| PAL1 | Os02g0626100 | phenylalanine ammonia-lyase gene | *M. Oryzae* | [175] |
| CAD2 | Os02g0187800 | Cinnamyl alcohol dehydrogenase | *M. Oryzae* | [176] |
| LAC7 | Os01g0850550 | Laccase | *M. Oryzae Xoo and R. Solani* | [158] |
| LAC28 | Os12g0257600 | Laccase | *M. Oryzae Xoo and R. Solani* | [158] |
| LAC29 | Os12g0258700 | Laccase | *M. Oryzae Xoo and R. Solani* | [158] |
| WAK5 | Os01g0364100 | Cell wall-associated KINASE | *M. Oryzae* | [93] |
| WAKL21.2 | Os12g0595800 | Receptor-like kinase | *Xoo* | [178] |
| WAK25 | Os03g0225700 | Wall-associated receptor-like kinase 25 | *M. Oryzae Xoo and R. Solani* | [179] |
| VTC1-1 | Os01g0847200 | Mannose-1-phosphate guanosine transferase | *M. Oryzae* | [180] |
| VQ8 | Os02g0539500 | Valine-glutamine motif containing protein | *M. Oryzae* and *Xoo* | [62] |
| GLP3-7 | Os03g0804500 | Germin-like protein | *M. Oryzae* and *Xoo* | [187] |
| WRKY10 | Os01g0186000 | WRKY transcription factor | *M. Oryzae* | [186] |
| NAC29 | Os08g0115800 | NAC Transcription Factor | *M. Oryzae* | [188] |
| TPS28 | Os07g0218200 | Ent-casbene synthase | *M. Oryzae* | [188] |
| CYP71Z2 | Os07g0217600 | Casbene C5 oxidase | *M. Oryzae* | [188] |
| STK | Os06g0209500 | TF of sugar signal transduction | *M. Oryzae* | [189] |
| NOMT | Os12g0240900 | Naringenin 7-O-methyltransferase | *M. Oryzae* | [189] |
